# Comparative Analysis of the Chloroplast Genomes of Cypripedium: Assessing the Roles of SSRs and TRs in the Non-Coding Regions of LSC in Shaping Chloroplast Genome Size

**DOI:** 10.3390/ijms26083691

**Published:** 2025-04-14

**Authors:** Huanchu Liu, Hans Jacquemyn, Yanlin Wang, Yuanman Hu, Xingyuan He, Ying Zhang, Yue Zhang, Yanqing Huang, Wei Chen

**Affiliations:** 1 Institute of Applied Ecology, Chinese Academy of Sciences, Shenyang 110016, China; 2 CAS Key Laboratory of Forest Ecology and Silviculture, Institute of Applied Ecology, Chinese Academy of Sciences, Shenyang 110016, China; 3 Shenyang Arboretum, Chinese Academy of Sciences, Shenyang 110016, China; 4 Department of Biology, Plant Conservation and Population Biology, KU Leuven, 3001 Leuven, Belgium; 5 College of Life Sciences, Shihezi University, Shihezi 832003, China

**Keywords:** *Cypripedium*, chloroplast genome, SSRs, TRs, Orchidaceae, phylogenomics

## Abstract

*Cypripedium* is renowned for its high morphological diversity and complex genetic and evolutionary characteristics. The chloroplast genome serves as a valuable tool for investigating phylogenetic relationships and evolutionary processes in plants. Currently, research on the evolution of the chloroplast genome within the *Cypripedium* genus is limited due to insufficient large-scale sampling and a lack of comprehensive understanding. Consequently, the mechanisms underlying the significant differences in chloroplast genome size among *Cypripedium* species remain poorly understood. In this study, we conducted a comprehensive comparative analysis of the chloroplast genomes of 29 *Cypripedium* species. The lengths of these genomes ranged from 162,092 to 246,177 base pairs (bp) and contained between 127 and 134 genes. Our results indicate that, while the overall structure of the chloroplast genomes in *Cypripedium* species is relatively conserved, significant differences were observed among the large single-copy (LSC), small single-copy (SSC), and inverted repeat (IR) regions. Several genes, including *psaC*, *rpl32*, *ycf1*, and *psbK*, exhibited higher levels of variability and may serve as molecular markers in taxonomic studies. The results of our correlation analysis suggest that the expansion of the LSC region, the increase in simple sequence repeats (SSRs), and tandem repeats (TRs) have significantly enlarged the size of the chloroplast genome in *Cypripedium* species. Phylogenetic signal testing supports the notion that genetic variation has driven species divergence within the genus. Overall, our findings provide insights into the substantial differences in chloroplast genome length observed among *Cypripedium* species. However, the relationship between diversification and the evolutionary mechanisms affecting *Cypripedium*, including ecological adaptive evolution, incomplete lineage sorting (ILS), hybridization, and reticulate events, requires further investigation.

## 1. Introduction

The Orchidaceae is one of the largest and most diverse families of flowering plants. Orchids are distributed worldwide except in polar regions and deserts [1]. Within the Orchidaceae, the genus *Cypripedium* is particularly renowned for its unique and highly ornamental flowers, which are admired for their distinct morphology [2]. Moreover, species of the genus *Cypripedium* show wide variation in distribution, as they are widely distributed across temperate regions of the Northern Hemisphere, while others have very restricted distribution areas inhabiting environments ranging from moist forests to arid grasslands and alpine areas [3,4]. Such diverse distribution patterns and habitat preferences make *Cypripedium* an ideal choice for investigating plant adaptive evolution and speciation.

Despite comprehensive phylogenetic investigations of Cypripedium spanning multiple molecular markers (from traditional nuclear ITS to advanced low-copy nuclear orthologs) [2,3,4,5,6,7,8,9,10], while the monophyly of the genus and majority of its sections is well established, substantial unresolved conflicts remain regarding the internal classification of Section *Cypripedium*. Most of these studies have employed a limited number of nuclear and chloroplast DNA markers, and only a few studies have focused on the chloroplast genome. The chloroplast genome is a crucial component of plant cells, primarily responsible for photosynthesis and other essential metabolic processes, such as the synthesis of amino acids and storage of carbohydrates and lipids [11]. The plastid genome is relatively small and highly conserved, typically consisting of a circular DNA molecule of 100–200 kilobases (kb) in size, with a ‘quadripartite’ structure consisting of two large inverted repeats (IRs) dividing the circle into a large single-copy region (LSC) and a small single-copy region (SSC) containing a range of genes involved in photosynthesis, protein synthesis, and various metabolic pathways [12]. Due to their conservation and maternal inheritance, chloroplast genomes hold significant value in plant phylogenetic and evolutionary studies [13,14,15].

Recent advances in high-throughput sequencing technology have facilitated the complete sequencing of numerous *Cypripedium* chloroplast genomes, yielding significant insights into their genomic evolution [6,7,8,16,17]. Research indicates that *Cypripedium* species possess the largest chloroplast genomes among orchids, with sizes ranging from 160 kb to 246 kb [7,8]. These plastomes encode 75–88 protein-coding genes, 8 transfer RNA genes, and in most cases, 38 ribosomal RNA genes (comprising duplicated copies of 4 distinct rRNA species) [7,8,10]. This considerable variation in genome size is likely driven by rapid evolutionary dynamics. Factors contributing to this genomic diversity include the presence of AT-rich repeat sequences, gene recombination, duplication, amplification, and loss [6,8]. Furthermore, Guo et al. [16] discovered that genes associated with floral fragrance and environmental adaptation may play a role in the species diversification of *Cypripedium*. Collectively, these elements influence the structural and functional variation of *Cypripedium* chloroplast genomes, reflecting their complex evolutionary history. However, most studies based on chloroplast genes have predominantly focused on closely related species within specific sections of the genus or on comparisons among a few sections, resulting in a lack of large-scale sampling and a comprehensive understanding of the evolution of the chloroplast genome in the genus *Cypripedium*. Moreover, previous research has predominantly focused on the sequence analysis of specific genes or gene fragments, as well as a limited number of complete chloroplast genomes [4,6,8,18,19]. While these analyses provide fundamental phylogenetic information, they may not fully capture the evolutionary history and genetic diversity of the genus *Cypripedium*. Data derived solely from gene fragments may lack the resolution necessary to distinguish closely related taxa [20], potentially overlooking critical variations and recombination events that are essential for understanding plant phylogenetics and evolutionary mechanisms. Many species of *Cypripedium* currently face extinction due to habitat loss, over-collection, and climate change [21,22]. Therefore, studying the structure and evolution of the chloroplast genome in *Cypripedium* is not only scientifically significant for understanding its evolutionary mechanisms and genetic diversity but also practically important for conservation and horticultural applications.

In this study, we compared the chloroplast genomes of 29 *Cypripedium* species to explore the structural variations and evolutionary dynamics within this genus. Through the sequencing and comparison of multiple *Cypripedium* chloroplast genomes, we aim to uncover variations in genome structure and their relevance to evolutionary history. Our objectives were the following: (1) characterize the size, GC content, and gene content of the chloroplast genomes across different *Cypripedium* species; (2) identify structural variations, such as inversions, expansions, and contractions, and their impact on genome evolution; (3) analyze the phylogenetic relationships among *Cypripedium* species based on chloroplast genome data and investigate the role of repeat sequences and nucleotide diversity in shaping these relationships. By addressing these objectives, we seek to provide a comprehensive understanding of the evolutionary mechanisms driving the diversification of *Cypripedium* chloroplast genomes.

## 2. Results

### 2.1. Chloroplast Genomes of Cypripedium

Across 29 *Cypripedium* species, chloroplast genome sizes ranged from 162,092 bp (*C. debile*) to 246,177 bp (*C. lichiangense*), with GC contents between 26% and 35.4%. All genomes displayed the typical quadripartite structure (LSC, SSC, and a pair of IRs). These species were assigned to 11 sections within Cypripedium, including *Cypripedium*, *Flabellinervia*, *Palangshanensia*, *Sinopedilum*, *Trigonopedia*, *Bifolia*, *Obusipetala*, *Wardiana*, *Subtropica*, *Arietinum*, and *Retinervia*.

Sectional averages of genome sizes varied from 177,497 to 238,054.5 bp. Six sections—including *Arietinum*, *Subtropica*, *Wardiana*, *Sinopedilum*, *Palangshanensia*, and *Trigonopedia*—had genomes larger than 200 kb. Interestingly, *C. lichiangense* had the largest genome but the lowest GC content, while *C. debile* had the smallest genome and highest GC content. Species in *Flabellinervia* and *Bifolia* exhibited relatively consistent genome metrics. Within Section *Cypripedium*, *C. ludlowii* had the longest genome (203,989 bp) and lowest GC content (29.6%), while others ranged from 175,122 to 200,587 bp with GC contents of 30–34.5% (Figure S1).

Among the species, *C. forrestii*, *C. formosanum,* and *C. tibeticum* contained the most genes (134), while *C. debile* and *C. elegans* had the fewest (128 and 127) (Table 1 and Supplementary Data File). The number of coding sequences (CDSs) of the 29 species ranges from 75 to 88, the number of pseudogenes ranges from 0 to 11, and all species contain 8 tRNA genes. Most species had 38 rRNA genes, except *C. formosanum* (39) and *C. micranthum* (37). There were 13 duplicated genes, including four rRNA genes and 9 other genes (such as *rpl2*, *rps19*, and various tRNA genes) (Table S2). Furthermore, we identified 6 intron-containing tRNA genes and 14 intron-containing protein-coding genes, including *trnA UGC*, *trnG-UCC*, *trnI GAU*, *trnK-UUU*, *trnL-UAA*, *trnV UAC*, *atpF*, *ndhA*, *ndhB*, *ndhC*, *petB*, *petD*, *rpl16*, *rpl1*, *rpl2*, *rpoC1*, *rps16*, *rps12*, *ycf3*, and *clpP*. Among then, *trnA UGC*, *trnI GAU*, *ndhB*, *ycf3*, and *clpP* each contained two introns (Table S3). The *clpP* intron is absent in *C. palangshanense* due to gene loss. Only the *ndhC* gene in *C. fargesii* contains an intron, as the *trnK-UUU* genes in *C. debile* and *C. palangshanense* have been lost, and the *rps16* genes in *C.* × *ventricosum* and *C*. *macranthos* are also absent, resulting in the lack of introns. The total length of introns ranges in 29 *Cypripedium* species from 20,435 bp (in *C. debile*) to 39,208 bp (in *C. subtropicum*), accounting for 12.6% to 18.4% of the total sequence length. The average intron length of all species was 14.3% of the sequence length.

### 2.2. Comparative Analysis of Chloroplast Genome Structure

**Figure 1:**
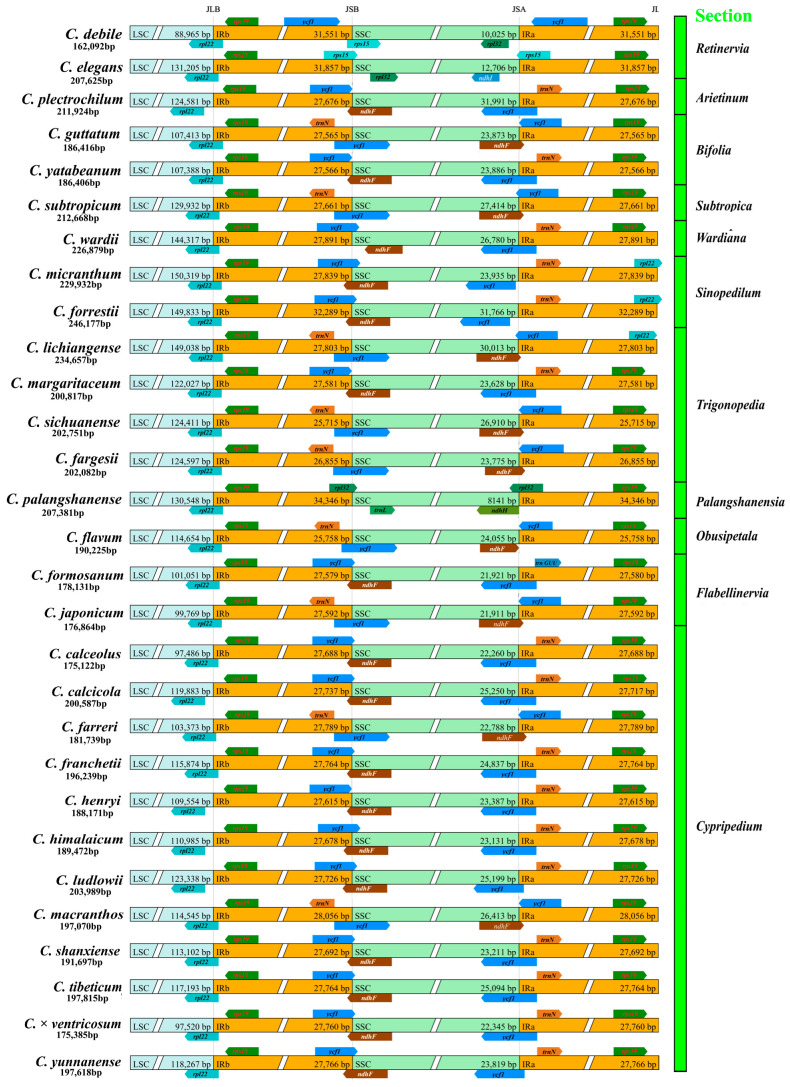
Comparison of the borders of the large single-copy region (LSC), small single-copy region (SSC), and inverted repeat (IR) regions of chloroplast genomes in 29 *Cypripedium* species.

The 29 *Cypripedium* species exhibited clear structural differences in the LSC, SSC, and IR regions. *C. micranthum* had the largest LSC (150,319 bp), while *C. debile* had the shortest LSC (89,446 bp). *C. palangshanense* had the largest IR region at 33,346 bp, but *C. sichuanense* had the shortest IR region (25,715), not *C. debile*, which had the shortest sequence overall. Despite its large genome size, *Cypripedium palangshanense* exhibited the shortest SSC at 8141 bp (see Figure 1 and Figure S2).

The *rps19* gene, located at the LSC/IRb boundary, ranged from 136 to 445 bp across all species examined. The LSC region consistently terminates with the *rpl22* gene in all species. In most species, the IRa/SSC boundary is crossed by the *ycf1* and *ndhF* genes, and the IRa region concludes with the *rps19* gene. However, in *C. guttatum*, *C. subtropicum*, *C. fargesii*, *C. sichuanense*, *C. lichiangense*, *C. flavum*, *C. japoncium*, *C. farreri*, and *C. macranthos*, the IR/SSC boundary contains the *trnN* gene. The IRa regions of *C. micranthum*, *C. forestii*, and *C. lichiangense* ends with the rpl22 gene. Notably, although the sequences of *C. debile*, *C. elegans*, and *C. palangshanense* are relatively short compared to other species, their *ycf1* genes are located within the IR region, which accounts for the extended IR regions observed in these three plants.

Genome alignment conducted using Mauve revealed significant large-scale inversions of approximately 80 kb in the LSC regions of *C. elegans*, *C. wardii*, *C. micranthum*, *C. forestii*, and most species from Section *Cypripedium* (*C. calceolus*, *C. calcicola*, *C. franchetii*, *C. himalaicum*, *C. ludlowii*, *C. shanxiense*, *C.* × *ventricosum*, and *C. yunnanense*). Although inversions have been observed in the SSC and IR regions across many species (e.g., *C. elegans*, *C. plectrochilum*, *C. yatabeanum*, *C. wardii*, and *C. calceolus*, etc.), this may be due to different researchers selecting different methods for unrolling the chloroplast genomes, rather than any substantial structural changes within the genomes themselves (Figure S3). Using *C. debile* as a reference, we compared the chloroplast genomes of 28 other species utilizing the mVISTA online tool. The positions of the variant genes were largely consistent with the locations of the inverted sequences identified in the Mauve analysis results. We observed significant variation in the LSC regions (including *atpA*, *atpF*, *rpoC1*, *rpoC2*, *rpoB*, and other genes) within the gene structures of *C. elegans*, *C. wardii*, *C. micranthum*, *C. forestii*, and most species from Section *Cypripedium*. In many species, such as *C. elegans*, *C. plectrochilum*, *C. yatabeanum*, *C. wardii*, *C. micranthum*, *C. forrestii*, *C. margaritaceum*, *C. palangshanense*, *C. formosanum*, and *C. calceolus*, the genes *ycf1, rpl32*, *ndhD*, and *ndhA* in the SSC region all showed large variation (Figure S4).

**Figure 2:**
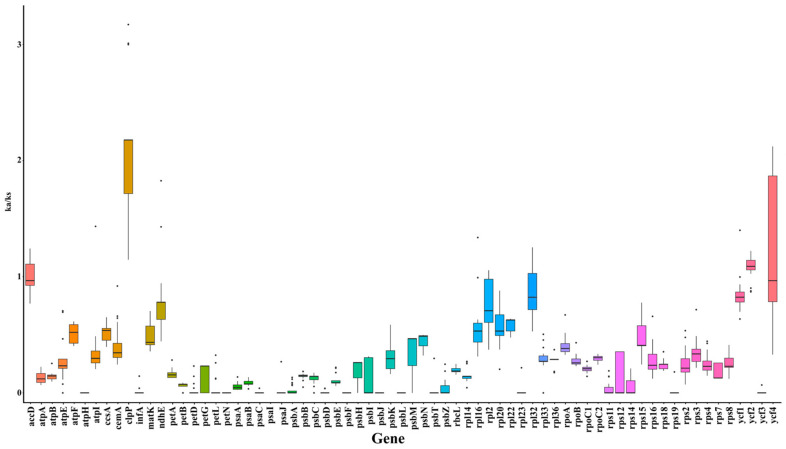
The Ka/Ks ratios for 69 genes across 28 species using *C. debile* as a reference.

To further investigate the evolutionary dynamics, the Ka/Ks ratios of 69 genes across 28 species were calculated using *C. debile* as a reference (Figure 2). The Ka/Ks ratio for the *clpP* gene exceeded 1 in all species, suggesting that this gene may be influenced by positive selection. Additionally, the Ka/Ks ratios for the *accD*, *ycf2*, and *ycf4* genes were greater than 1 in most species, indicating that these genes may play a significant role in the mutational evolution of species. The nucleotide diversity (Pi) of the 69 genes ranged from 0.00079 to 0.09277, with an average of 0.0094. The Pi values observed in the *psaC*, *rpl32*, *ycf1*, and *psbK* regions were greater than 0.2, suggesting that these regions may be under selective pressure (Figure S5).

### 2.3. Repeat Sequence Analysis

**Figure 3:**
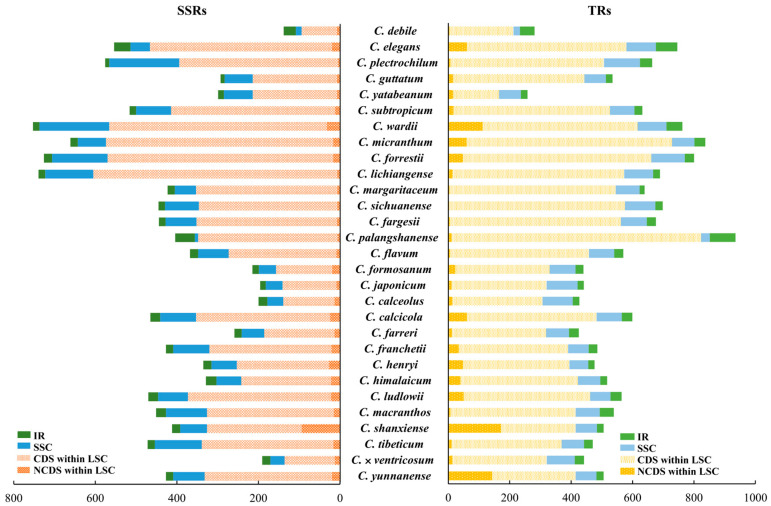
Statistics of SSRs and TRs in IR, SSC, the coding regions (CDSs) within LSC, and non-coding sequence (NCDS) within LSC in 29 *Cypripedium* species.

We identified 12,723 simple sequence repeats (SSRs) in the chloroplast genomes of 29 *Cypripedium* species, averaging 439 SSRs per species. Additionally, we found 16,802 tandem repeats (TRs), with an average of 579 per species. The highest number of SSRs was observed in *C. lichiangense* (781), and the lowest was found in *C. debile* (139) (Figure S6 and Table S4). The most common SSRs motifs were A/T and AT/AT, indicating a high prevalence of poly-A and poly-T sequences, whereas poly-C and poly-G motifs were relatively rare. The five most common types of SSRs were A/T, AT/TA, AAT/ATT, AAAT/ATTT, and AATAT/ATATT, which together accounted for 87.5% of the total (Figure S5). Notably, species with longer genomes, such as *C. wardii*, *C. lichiangense*, *C. forrestii*, and *C. micranthum*, have the longest sequences and the highest counts of SSRs, all exceeding 700. However, *C. forrestii*, *C. micranthum*, and *C. palangshanense* have the most TRs, each exceeding 800. Among these, *C. forrestii*, which has the longest sequence, does not possess the highest counts of SSRs and TRs, but the combined total of both is the highest. The statistics of SSRs and TRs presented in Figure 3 indicate that LSC regions, particularly the non-coding regions within the LSC, contain a significant number of SSRs and TRs, accounting for 77.3% of all repeats (Figure 3).

### 2.4. Gene Length Correlations 

The results of the Pearson correlation analysis are presented in Table S5. The analysis revealed that gene length was significantly positively correlated with LSC length (r = 0.98, *p* < 0.01), non-coding sequence (NCDS) length (r = 0.98, *p* < 0.01), SSRs (r = 0.96, *p* < 0.01), TRs (r = 0.83, *p* < 0.01), and intron length (r = 0.85, *p* < 0.01). Additionally, most SSRs and TRs occurred in the LSC region (see Figure 3). In contrast, a significant negative correlation was observed with GC content (r = −0.96, *p* < 0.01), particularly with the GC content of the LSC (r = −0.96, *p* < 0.01). No significant correlations (*p* > 0.05) were found between gene length variation and gene number, IR length, or coding sequence length. These findings suggest that the expansion of the LSC region and non-coding sequence length and the increase in SSRs and TRs, as well as the intron length, have contributed to the overall increase in gene length.

### 2.5. Phylogenetic Relationships

**Figure 4:**
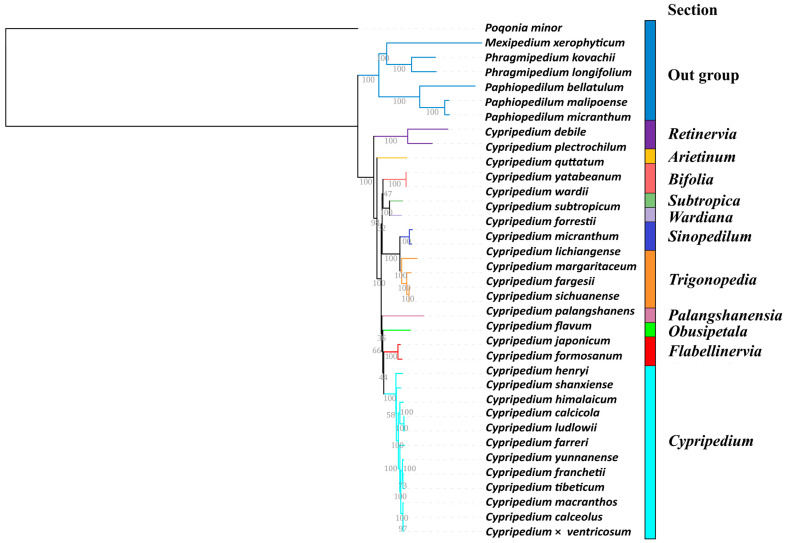
Phylogram obtained from Maximum Likelihood (ML) analysis of the coding sequence regions of the chloroplast data obtained from 29 *Cypripedium* species, 6 species from related groups and *Pogonia minor*. Numbers at the nodes are bootstrap percentages.

The phylogenetic analysis of the CDS regions, comprising a 55,503 base pair alignment matrix from 36 orchid chloroplast genomes, was conducted using Maximum Likelihood (ML) estimation. This analysis received strong support for most evolutionary branches (see Figure 4). The results provide robust evidence for the monophyly of the subfamily Cypripedioideae and the genus *Cypripedium*.

Within the subfamily Cypripedioideae, the genera *Mexipedium*, *Phragmipedium*, and *Paphiopedilum* are grouped into a well-supported evolutionary clade, with *Mexipedium* and *Phragmipedium* forming sister clades. The 29 species of the genus *Cypripedium* are divided into eight major evolutionary clades. The sections *Retinervia* and *Arietinum* are identified as early-diverging lineages, with bootstrap support values of 100% and 98%, respectively. The Section *Trigonopedia*, which includes species such as *C. lichiangense*, *C. fargesii*, *C. margaritaceum*, and *C. sichuanense*, forms a monophyletic group that clusters with *Sinopedilum* into a well-supported evolutionary branch (100% bootstrap support). This branch is sister to the common ancestor of the sections *Subtropica*, *Wardiana*, and *Bifolia*. The sections *Palangshanensia* and *Obusipetala* are independent evolutionary branches, but they exhibit lower support values (36% bootstrap support), indicating potential ambiguity in their relationships. Simultaneously, the sections *Cypripedium* and *Flabellinervia*, which include species like *C. formosanum* and *C. japonicum*, are derived from a common ancestor and are confirmed as monophyletic groups. Within the Section *Cypripedium*, 19 species are further divided into six evolutionary branches. Notably, *C. henryi* and *C. shanxiense* are identified as early-diverging lineages within this branch. The species *C. himalaicum*, *C. calcicola*, and *C. ludlowii* form an evolutionary clade. The common ancestor of *C. macranthos*, *C*. *calceolus*, and *C*. × *ventricosum* is closely related to the common ancestor of *C. yunnanense*, *C. franchetii*, and *C. tibeticum*.

### 2.6. Phylogenetic Signal Analysis

The results of the phylogenetic signal test are presented in Table 2 and Table S6. The evolution of total length, LSC length, SSC length, IR length, CDS length, NCDS length, SSRs, TRs, and intron length exhibited significant phylogenetic signals (*p* < 0.01), with their lambda values equal to or approaching 1. This indicates that the variations in these traits are closely associated with phylogeny. Although Blomberg’s K for these variables was significant, it was relatively low, ranging from 0.141 to 0.518 overall. This suggests that the similarity of these traits among closely related species is lower than anticipated.

## 3. Discussion

### 3.1. Characterization of Organellar Genomes

The chloroplast genomes of *Cypripedium* species exhibit significant variation in length, ranging from 162,773 bp to 246,177 bp, with *C. forrestii* possessing the largest chloroplast genome not only within the genus but also among all Orchidaceae species. In contrast to the relatively conserved chloroplast genomes found in other orchid genera, such as *Paphiopedilum*, *Pholidota*, and *Phalaenopsis* [6,23,24], the expansion and contraction of boundary regions between SC and IR regions commonly account for these differences in chloroplast genome length [25]. Furthermore, our study indicates that the occurrence of large-scale inversions in *Cypripedium* is not entirely independent. The two species in the *Sinopedilum*, which possess the longest chloroplast genomes in the genus, all underwent large-scale inversions, suggesting that these inversions may have occurred prior to the divergence of this section. Similar inversions have been observed in eight of the 12 species in the Section *Cypripedium*; however, these species do not cluster together, suggesting that their inversions may have occurred independently. These large-scale chromosomal inversions are also found in Paphiopedilum species [26], suggesting that structural variations such as inversions, translocations, and duplications play a crucial role in species differentiation [27].

The number of genes in the 29 *Cypripedium* species ranges from 127 to 134. Significant changes have been observed in the SC/IRb boundary among different sections of the *Cypripedium* genus, whereas boundary variations among species within the same section are relatively minor. The SSC region of the early group *Retinervia* is short, averaging 11,365 bp. Subsequently, with the exception of *C. panlangshens*, whose SSC length is only 8142 bp, the SSC of other species has expanded to varying degrees, exceeding 20 kb in length. Chloroplast quadripartite structure variation may be closely related to phylogenetic relationships, a finding that is further supported by the results of phylogenetic signal detection. We observed the loss of the *matK* gene in *C. plectrochilum*, *C. henryi*, *C. palangshanense*, *C.flavum*, and *C. subtropicum*, and Guo et al. [6] suggested that *matK* in this genus may be transitioning to a pseudogene, with other mechanisms potentially compensating for the splicing of group IIA introns. Notably, *C. subtropicum* is unique in exhibiting the loss of the *trnK* gene and the duplication of the *trnL* gene. Overall, the loss or pseudogenization of *ndh* genes is a common phenomenon in the chloroplast genomes of Orchidaceae, particularly among epiphytic orchids, although it occasionally occurs in the subfamily Cypripedioideae [28]. The region extending from the IRb to the SSC exhibits the highest nucleotide variability, which may be associated with the expansion and contraction of IR regions and the transfer of *ndh* genes from the chloroplast to the mitochondrial genome [8].

### 3.2. The Expansion and Contraction of Organellar Genomes

Future research directions may also be highlighted. Repeated sequences significantly influence the structure and sequence variation of chloroplast genomes [29,30]. Variations in the number of simple sequence repeats (SSRs) within chloroplast genomes serve as key molecular markers, referred to as cpSSRs, and are widely utilized in population genetics, polymorphism studies, and evolutionary research within the Orchidaceae family [31,32,33,34,35]. Consistent with the findings of Liu et al. [36] and Tao et al. [24] in other Orchidaceae species, the chloroplast genomes of *Cypripedium* exhibit a significantly higher content of A/T repeats compared to G/C repeats. However, unlike other genera such as *Phalaenopsis*, *Neottianthe*, *Ponerorchis*, *Polystachya*, *Coelogyne*, and *Otochilus* [24,36,37], where A/T repeats are overwhelmingly dominant, *Cypripedium* species (including *C. micranthum*, *C. fargesii*, *C. sichuanense*, *C. lichiangense*, and *C. subtropicum*) particularly feature AT/TA and AAT/ATT repeats. This phenomenon may require further validation regarding its connection to specific genome replication events, changes in mutation rates, and genetic drift. The proliferation of satellite DNA is one of the important mechanisms of DNA accumulation [38,39], which may act as an evolutionary ‘tuning knob’ and play a crucial role in generating genetic variation that underlies adaptive evolution [40]. A strong correlation was observed between sequence length in *Cypripedium* species and a preference for A/T repeats. Species with longer sequences tend to exhibit significantly lower GC content and a higher abundance of AT, AT/TA, and AAT/ATT repeats, with these three types of repeats accounting for 52% of the total repeats, consistent with the findings of Guo et al. in *C. tibeticum* and *C. subtropicum* [6]. Tandem repeats play an important role in causing plastid genome mutations [41] and are one of the important types of repeats in *Cypripedium*. Compared with other genera in Cypripedioideae, SSR and TF repeats in *Cypripedium* species have also undergone significant expansion (Table S7). Our results also confirmed that they are not only significantly correlated with sequence expansion but also closely related to phylogenetic relationships.

The expansion and contraction of the IR (25,715–34,346 bp), SSC (8141–31,991 bp), and LSC (88,965–150,319 bp) regions in *Cypripedium* species highlight the complex evolutionary history of this genus. Our findings indicate that the expansion of the LSC region significantly contributes to the overall increase in genome size, as it constitutes the largest component of the quadripartite structure of the chloroplast genome, accounting for 55% to 62.2% of the total length. The LSC region also exhibits greater variability and more dynamic evolutionary behavior, likely due to the presence of fewer conserved genes and regions [42]. Similarly, *Cypripedium* species display significant variation in their nuclear genomes, with genome sizes ranging from 4.14 pg to 44.84 pg, representing an 11-fold difference (https://cvalues.science.kew.org/, 21 January 2025). Despite this variation, chromosome numbers remain relatively stable across the genus, with most species possessing 2n = 20 chromosomes [43]. An exception is *C. macranthos*, which displays the widest chromosomal variation within the genus, with counts of 2n = 20, 21, 30, and 36 [3]. Leitch et al. hypothesized that the ancestral *Cypripedium* genome was smaller, with subsequent species exhibiting an increase in genome size [44]. Although the chloroplast genomes of *Cypripedium* are unusually variable compared to other plant genera, current evidence suggests there is no clear correlation between nuclear and chloroplast genome sizes within the genus. For example, members of Section *Cypripedium*, which represent later-diverging lineages, tend to have larger nuclear genomes but do not necessarily possess the largest chloroplast genomes. Our findings indicate that increased SSRs and TRs, intron length, and LSC region expansion are significant mechanisms driving the enlargement of chloroplast genome size among *Cypripedium* species.

### 3.3. Phylogenetic Relationships in Organellar Genomes

Phylogenetic trees based on nuclear genomes integrate genetic contributions from both parents; however, plant nuclear genomes are highly complex [45]. In contrast, chloroplast genomes represent a distinct genetic system [46] and generally reflect only the maternal lineage, which often leads to inconsistencies with phylogenies based on nuclear genomes. Although this study exclusively sampled *Cypripedium* species, primarily from East Asia, our phylogenetic results align with those of Li et al. [19] and Liao et al. [7], who identified *Retinervia* as part of an early-diverging lineage in Asia. However, these findings differ from those of Chen et al. [3] and Liu et al. [4], who proposed that *C. subtropicum* represents the earliest lineage in East Asia. Nevertheless, all studies concur that the sections *Trigonopedia* and *Sinopedilum* are monophyletic and form sister clades. These two sections share several key traits, including two leaves, single flowers, true bracts, and a pedicel that elongates after fertilization [3].

It is well established that the Section *Cypripedium* is monophyletic. However, the evolutionary relationships among species within this section remain unresolved. For instance, instead of clustering with *C. calceolus*, which has traditionally been regarded as a sister species, *C. shanxiesne* is an early group within the Section *Cypripedium* alongside *C. henryi*. Notably, the labellum of both species is deeply sac-oval and nearly identical in size. Previous studies [47,48,49] and our observations suggest that widespread hybridization occurs among co-occurring species within the section. Additionally, complex selective pressures and recombination events likely influence the phylogenetic outcomes [50,51,52]. The results of the phylogenetic signal detection analyses indicate a close relationship between the evolution of certain genetic traits and phylogenetic relationships. However, the relatively low K values suggest that these traits exhibit less similarity among closely related species than anticipated. This implies that species may have undergone rapid adaptations to various environmental pressures, resulting in significant variation that exceeds what would be expected based solely on phylogeny. Furthermore, recent evidence supports the hypothesis that incomplete lineage sorting (ILS), resulting from rapid radiation and compounded by multiple potential reticulate events, has contributed to the diversification of *Cypripedium* [17].

## 4. Materials and Methods

### 4.1. Data Acquisition

We retrieved all available complete chloroplast genomes of the *Cypripedium* species from the NCBI database. As of 31 December 2024, genome sequences for 29 species were publicly available. Due to the AT-rich nature of *Cypripedium* species, second-generation sequencing may overlook certain regions with high AT content [6]. Therefore, for species that each have multiple complete sequences, we prioritized reference genomes generated by combining both second- and third-generation sequencing data. For each species, only the most reliable complete sequence was retained (see Table 1 for species and sequence details). These sequences were manually inspected using Geneious Prime v. 2021.1 (Biomatters Ltd., Auckland, New Zealand) to identify potential annotation omissions or errors. We first standardized gene names to ensure consistency across all annotations. Subsequently, we verified the start and stop codons of protein-coding genes, corrected incorrectly annotated intron–exon boundaries, and adjusted gene orientations when necessary. Each chloroplast genome was then compared with those of closely related *Cypripedium* species to recover any potentially missing genes. Additionally, species-specific genes were manually examined to assess whether they represent genuine genomic features or annotation artifacts resulting from automated prediction processes.

### 4.2. Comparison of Chloroplast Genome Sequences

The boundaries between the IR, SSC, and LSC regions of the chloroplast genomes were compared using CPJSdraw software v. 0.0.1 [53]. Chloroplot was used for diverse visualization of organellar genomes [54]. The chloroplast genomes were aligned and visualized with mVISTA (https://genome.lbl.gov/vista/mvista/submit.shtml, accessed on 13 January 2025) [55], using *C. debile* as the reference sequence. To identify regions of high variability, multiple genome alignments were performed with MAFFT v7.475 under default settings [56]. Polymorphic sites and nucleotide variability (Pi) were assessed using a sliding window approach with a window size of 600 base pairs (bp) and a step size of 200 bp, implemented in an online tool (http://112.86.217.82:9929/#/tool/alltool/detail/328, accessed on 13 January 2025). Repetitive sequences were identified using the Repeat Finder plugin (https://www.geneious.com/plugins/repeat-finder, accessed on 15 January 2025) in Geneious Prime. Gene arrangements and structural variations were further visualized and analyzed using Mauve software (https://darlinglab.org/mauve/mauve.html, accessed on 15 January 2025). In Geneious, after identifying the IR regions using plugins, the starting points of all sequences were adjusted to the first base of the single-cop region. Python v. 3.13.3 scripts were employed to extract CDS for all species, and annotation files were utilized to identify genes containing introns and to measure intron length. Ka/Ks ratios were calculated using KaKs_Calculator 2.0 to investigate the selective pressures acting on plastid protein-coding genes across the 29 *Cypripedium* species [57].

### 4.3. Analysis of Repeat Sequences

SSRs (≥10 bp) were identified using the MISA tool v2.1 [58]. The minimum thresholds were established at 10, 5, 4, 3, 3, and 3 for mononucleotides, dinucleotides, trinucleotides, tetranucleotides, pentanucleotides, and hexanucleotides, respectively. TRs were identified using the default parameters of the TRF tool (https://github.com/Adamtaranto/TRF2GFF, accessed on 15 January 2025). To exclude the interference of microsatellite repeats on TR results, we set the minsize to 7. To compare repeat sequences across the LSC, SSC, and IR regions, SSRs and TRs analyses were performed for each region of each species, and the total number of repeats was summarized for each region.

### 4.4. Correlation Analysis

Given the significant differences in the total chloroplast genome size among different *Cypripedium* species, we performed a Pearson correlation analysis of the total genome length and various genomic variables using SPSS Statistics 22.0. Initially, we conducted statistical analyses on GC content, LSC length, SSC length, IR length, number of genes, CDS length, and NCDS length, as well as SSRs and TRs. Considering that introns are non-coding DNA sequences that are spliced during gene expression, they are part of the complete chloroplast genome and may play a role in plant gene regulation and evolutionary adaptation. Therefore, we performed a statistical analysis of intron length for each species and incorporated these data into our correlation analysis.

Given the strong correlation between total length, LSC length, and repeats (SSRs and TRs), we further analyzed the distribution of SSRs and TRs in LSC, SSC, and IR regions. Specifically, the correlation analysis included the following 20 variables: total length, GC content, number of genes, LSC length, SSC length, IR length, CDS length, NCDS length, GC content of LSC, GC content of SSC, GC content of IR, SSRs, SSRs in LSC, SSRs in SSC, SSRs in IR regions, TRs, TRs in LSC, TRs in SSC, TRs in IR, and intron length. The goal of this analysis was to determine whether repeats in the LSC region contribute to genome expansion. Additionally, we extracted the start and end points of the LSC coding region and, based on the results from MISA and TRF, conducted a detailed comparison of the repeat sequence counts between the LSC coding and non-coding regions.

### 4.5. Phylogenetic Analysis

To investigate the phylogenetic relationships between *Cypripedium* and other genera within the subfamily Cypripedioideae, we included six additional species from the genera *Mexipedium*, *Phragmipedium*, and *Paphiopedium*, with *Pogonia minor* serving as the outgroup (Table S1). To minimize the potential interference from repeat sequences and non-coding regions, we constructed the phylogenetic tree using only the coding sequences (CDSs) of all species. Coding regions are typically more conserved and subject to stronger selective pressures, providing more stable and reliable phylogenetic signals. First, the CDS sequences for all species were extracted and aligned using MAFFT with default parameters. After alignment, the CDS of each species were concatenated. Phylogenetic analysis was then performed using RAxML v8.2.12 [59], and the best-fitting nucleotide substitution model was selected using jModelTest2 (https://github.com/ddarriba/jmodeltest2, accessed on 18 January 2025).

### 4.6. Phylogenetic Signal Test

To assess whether variations in chloroplast genomes, such as total length, GC content, number of genes, LSC length, SSC length, IR length, CDS length, NCDS length, GC content of LSC, GC content of SSC, GC content of IR, SSRs, SSRs in LSC, SSRs in SSC, SSRs in IR, TRs, TRs in LSC, TRs in SSC, TRs in IR, and intron length, exhibit a phylogenetic signal in *Cypripedium*, we utilized functions from the R packages ‘Phytools’ (version 0.3-93) and ‘geiger’ to calculate Pagel’s lambda (λ) and Blomberg’s *K* [60,61,62,63]. Pagel’s lambda evaluates the extent to which the evolution of a specific trait corresponds with the phylogenetic tree, while Blomberg’s *K* compares the observed variation in a trait among species to the variation expected under a Brownian motion model of evolution along the tree. The phylogenetic tree employed in this study was constructed based on the maximum likelihood topology derived from the complete chloroplast genomes of 29 *Cypripedium* species. However, due to the low variance in certain genomic features (e.g., GC and number of genes), the covariance matrix became singular, hindering reliable estimation of the phylogenetic signal for these variables. To address this issue, we excluded these highly conserved traits from the phylogenetic signal analysis.

## 5. Conclusions

This study provides a comprehensive comparative analysis of the chloroplast genomes across multiple *Cypripedium* species and shows significant variation in genome size compared to the more conserved chloroplast genomes found in other orchid genera. The contributors to this structural variation are the expansion and contraction of the SC and IR regions. Furthermore, species with longer genomes tend to exhibit a higher number of SSRs, particularly AT/TA and AAT/ATT repeats. The increase in SSRs and TR, along with the expansion of the LSC region significantly contributes to the elongation of the chloroplast genome. Phylogenetic signal tests further support the close association between genetic traits, such as TF and IR length, and species diversification within the genus. Overall, this study enhances our understanding of the evolutionary mechanisms that shape the chloroplast genome in *Cypripedium* and provides valuable insights for future research. Follow-up studies should expand the scope by including a broader range of species and incorporating functional genomics approaches to more fully elucidate the phylogenetic relationships and evolutionary mechanisms driving this diverse genus.

## Figures and Tables

**Table 1:** General characteristics of the chloroplast genomes of 29 *Cypripedium* species.

Species	Total Length (bp)	GC Content (%)	Number of Genes	Protein Coding Genes	tRNA Genes	rRNA Genes	Pseudogenes	GenBank No.
*C. debile*	162,092	35.4	128	76	8	38	6	PP811663
*C. elegans*	207,625	27.7	127	81	8	38	2	OR698927
*C. plectrochilum*	211,924	28.4	133	85	8	38	2	PP811672
*C. guttatum*	186,416	32.4	133	86	8	38	1	PP811666
*C. yatabeanum*	186,406	32.4	133	86	8	38	1	PP811674
*C. subtropicum*	212,668	28.2	132	86	8	38	0	MT937100
*C. wardii*	226,879	26.4	131	85	8	38	0	OR698942
*C. micranthum*	229,932	26.7	133	88	8	37	0	OR698938
*C. forrestii*	246,177	26.7	134	88	8	38	0	OR698931
*C. lichiangense*	234,657	26	132	86	8	38	1	NC_084419
*C. margaritaceum*	200,817	29.6	130	77	8	38	7	PP811670
*C. sichuanense*	202,751	29.1	133	82	8	38	5	PP811673
*C. fargesii*	202,082	29.2	132	86	8	38	0	NC_084418
*C. palangshanense*	207,381	29.2	130	77	8	38	7	PP811671
*C. flavum*	190,225	30.4	132	75	8	38	11	PP811665
*C. formosanum*	178,131	33.9	134	87	8	39	0	NC_026772
*C. japonicum*	176,864	34.1	133	85	8	38	2	PP811668
*C. calceolus*	175,122	34.4	133	87	8	38	0	NC_045400
*C. calcicola*	200,587	30	133	87	8	38	0	OR698926
*C. farreri*	181,739	33.1	133	86	8	38	1	PP811664
*C. franchetii*	196,239	30.7	133	87	8	38	0	OR698932
*C. henryi*	188,171	32	133	85	8	38	2	PP811667
*C. himalaicum*	189,472	31.8	133	87	8	38	0	OR698935
*C. ludlowii*	203,989	29.6	133	87	8	38	0	OR698937
*C. macranthos*	197,070	30.6	133	86	8	38	1	PP811669
*C. shanxiense*	191,697	31.5	133	87	8	38	0	OR698940
*C. tibeticum*	197,815	30.5	134	88	8	38	0	NC_053552
*C. × ventricosum*	175,385	34.5	131	85	8	38	0	PP448181
*C. yunnanense*	197,618	30.6	133	87	8	38	1	OR698943

**Table 2:** Phylogenetic signal tests for total length, LSC length, SSC length, IR length, coding sequence (CDS) length, non-coding sequence (NCDS) length, simple sequence repeats (SSRs), tandem repeats (TRs), and intron length using Pagel’s lambda and Blomberg’s K for 29 *Cypripedium* species. GC content (GC) and number of genes were excluded due to highly conserved features of the data. Variables with significant phylogenetic signals are indicated by * (*p* < 0.05) and ** (*p* < 0.01).

Variables	Total Length	LSC Length	SSC Length	IR Length	CDS Length	NCDS Length	SSRs	TRs	Intron Length
K	0.24 **	0.27 **	0.33 **	0.44 **	0.34 **	0.24 **	0.14 *	0.52 **	0.15 **
lambda	0.90 **	0.92 **	0.90 **	0.89 **	0.95 **	0.88 **	0.86 **	0.95 **	0.93 **

## Data Availability

The data supporting the conclusions of this article will be made available by the authors on request.

## Supplementary Materials

The following supporting information can be downloaded at: https://www.mdpi.com/article/10.3390/ijms26083691/s1.

## Abbreviations

The following abbreviations are used in this manuscript:

LSC	Large single-copy region
SSC	Small single-copy region
IR	Inverted repeat
CDS	Coding sequence
NCDS	Non-coding sequence
SSRs	Simple sequence repeats
TRs	Tandem repeats (TRs)
