# Supplementary material for: Comparative Analysis of the Chloroplast Genomes of Cypripedium: Assessing the Roles of SSRs and TRs in the Non-Coding Regions of LSC in Shaping Chloroplast Genome Size
Source: Int J Mol Sci. 2025 Apr 14;26(8):3691. doi: 10.3390/ijms26083691 (PMC12027508; doi:10.3390/ijms26083691)
Supplement: Supplementary file 1 [file ijms-26-03691-s001.zip › C. franchetii.pdf]

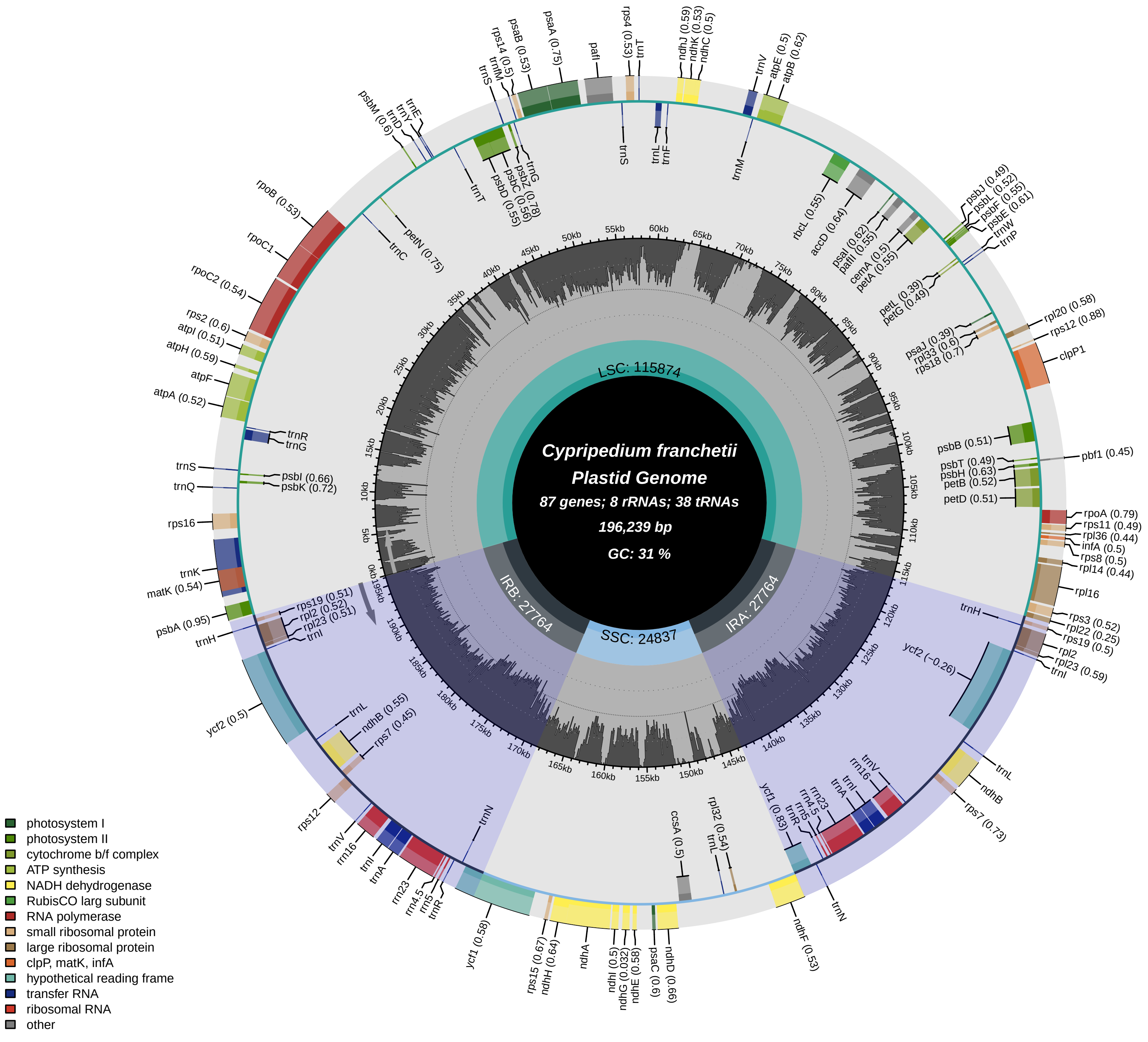
Cypripedium franchetii
Plastid Genome
87 genes; 8 rRNAs; 38 tRNAs
196,239 bp
GC: 31 %
LSC: 115874
IRA: 27764
SSC: 24837
IRB: 27764
psbA (0.95)
trnH
matK (0.54)
trnK
rps16
trnQ
trnS
psbI (0.66)
psbK (0.72)
trnR
trnG
atpA (0.52)
atpF
atpH (0.59)
atpI (0.51)
rps2 (0.6)
rpoC2 (0.54)
rpoC1
rpoB (0.53)
trnC
petN (0.75)
psbM (0.6)
trnD
trnY
trnE
trnT
psbD (0.55)
psbC (0.56)
psbZ (0.78)
trnG
trnS
trnfM
rps14 (0.5)
psaB (0.53)
psaA (0.75)
pafI
rps4 (0.53)
trnT
trnS
trnL
trnF
ndhJ (0.59)
ndhK (0.53)
ndhC (0.5)
trnV
atpE (0.5)
atpB (0.62)
trnM
rbcL (0.55)
accD (0.64)
psaI (0.62)
pafII (0.55)
cemA (0.5)
petA (0.55)
psbJ (0.49)
psbL (0.52)
psbF (0.55)
psbE (0.61)
trnW
trnP
petL (0.39)
petG (0.49)
psaJ (0.39)
rpl33 (0.6)
rps18 (0.7)
rpl20 (0.58)
rps12 (0.88)
clpP1
psbB (0.51)
pbf1 (0.45)
psbT (0.49)
psbH (0.63)
petB (0.52)
petD (0.51)
rpoA (0.79)
rps11 (0.49)
rpl36 (0.44)
infA (0.5)
rps8 (0.5)
rpl14 (0.44)
rpl16
trnH
rps3 (0.52)
rpl22 (0.25)
rps19 (0.5)
rpl2
rpl23 (0.59)
trnI
ycf2 (−0.26)
trnL
ndhB
rps7 (0.73)
trnV
rrn16
trnI
trnA
rrn23
rrn4.5
rrn5
trnR
ycf1 (0.83)
trnN
ndhF (0.53)
rpl32 (0.54)
trnL
ccsA (0.5)
ndhD (0.66)
psaC (0.6)
ndhE (0.58)
ndhG (0.032)
ndhI (0.5)
ndhA
ndhH (0.64)
rps15 (0.67)
ycf1 (0.58)
trnN
trnR
rrn5
rrn4.5
rrn23
trnA
trnI
rrn16
trnV
rps12
ycf2 (0.5)
trnL
ndhB (0.55)
rps7 (0.45)
rps19 (0.51)
rpl2 (0.52)
rpl23 (0.51)
trnI
0kb
5kb
10kb
15kb
20kb
25kb
30kb
35kb
40kb
45kb
50kb
55kb
60kb
65kb
70kb
75kb
80kb
85kb
90kb
95kb
100kb
105kb
110kb
115kb
120kb
125kb
130kb
135kb
140kb
145kb
150kb
155kb
160kb
165kb
170kb
175kb
180kb
185kb
190kb
195kb
photosystem I
photosystem II
cytochrome b/f complex
ATP synthesis
NADH dehydrogenase
RubisCO larg subunit
RNA polymerase
small ribosomal protein
large ribosomal protein
clpP, matK, infA
hypothetical reading frame
transfer RNA
ribosomal RNA
other
