# Supplementary material for: Comparative Analysis of the Chloroplast Genomes of Cypripedium: Assessing the Roles of SSRs and TRs in the Non-Coding Regions of LSC in Shaping Chloroplast Genome Size
Source: Int J Mol Sci. 2025 Apr 14;26(8):3691. doi: 10.3390/ijms26083691 (PMC12027508; doi:10.3390/ijms26083691)
Supplement: Supplementary file 1 [file ijms-26-03691-s001.zip › C. japonicum.pdf]

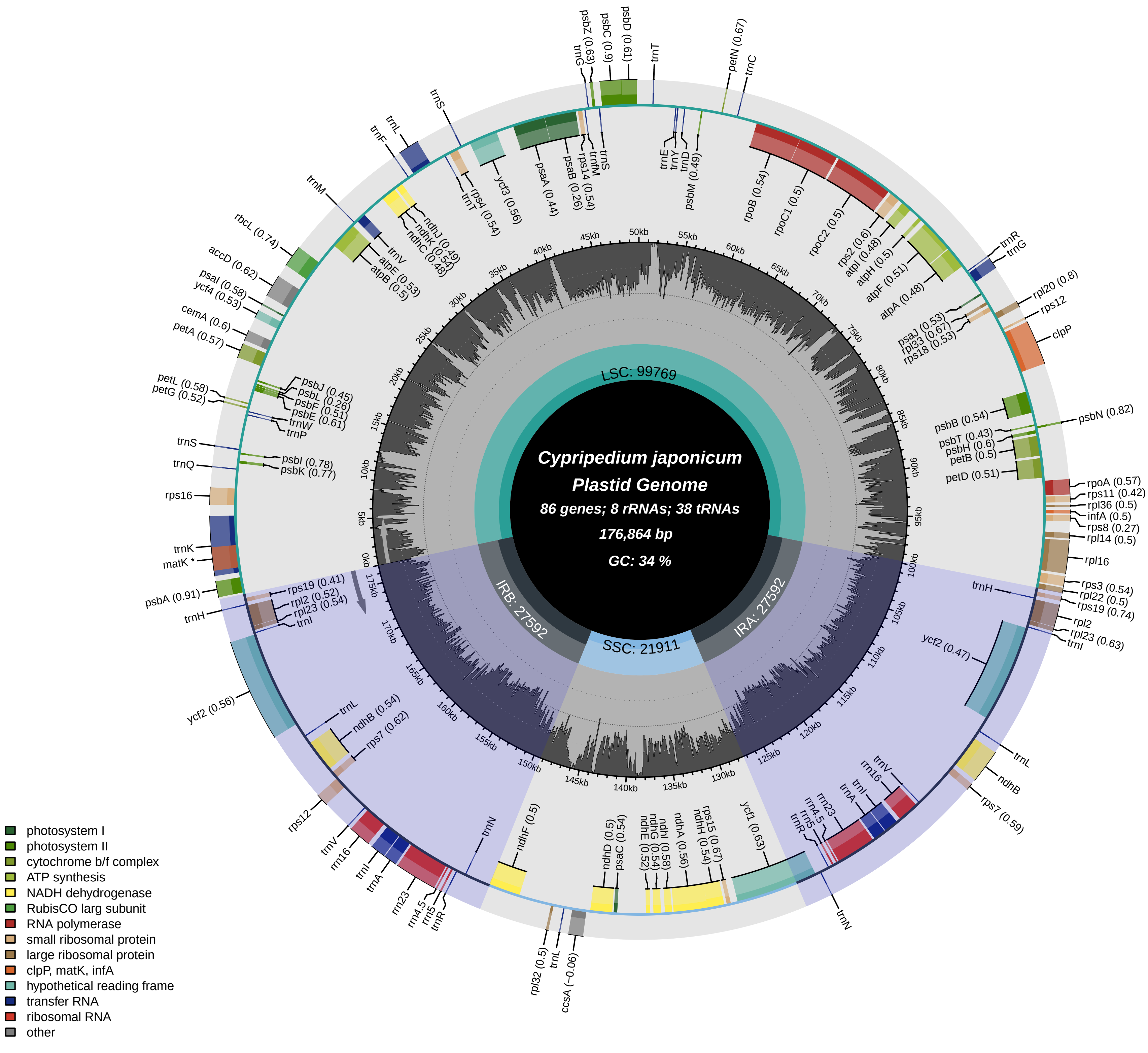
Cypripedium japonicum
Plastid Genome
86 genes; 8 rRNAs; 38 tRNAs
176,864 bp
GC: 34 %
LSC: 99769
IRA: 27592
SSC: 21911
IRB: 27592
photosystem I
photosystem II
cytochrome b/f complex
ATP synthesis
NADH dehydrogenase
RubisCO larg subunit
RNA polymerase
small ribosomal protein
large ribosomal protein
clpP, matK, infA
hypothetical reading frame
transfer RNA
ribosomal RNA
other
trnG
psbZ (0.63)
psbC (0.9)
psbD (0.61)
trnT
petN (0.67)
trnC
trnS
trnL
trnF
trnM
rbcL (0.74)
accD (0.62)
psaI (0.58)
ycf4 (0.53)
cemA (0.6)
petA (0.57)
petL (0.58)
petG (0.52)
psbJ (0.45)
psbL (0.26)
psbF (0.51)
psbE (0.61)
trnW
trnP
trnS
trnQ
psbI (0.78)
psbK (0.77)
rps16
trnK
matK *
psbA (0.91)
trnH
rps19 (0.41)
rpl2 (0.52)
rpl23 (0.54)
trnI
ycf2 (0.56)
trnL
ndhB (0.54)
rps7 (0.62)
rps12
trnV
rrn16
trnI
trnA
rrn23
rrn4.5
rrn5
trnR
trnN
ndhF (0.5)
rpl32 (0.5)
trnL
ccsA (−0.06)
ndhD (0.5)
psaC (0.54)
ndhE (0.52)
ndhG (0.54)
ndhI (0.58)
ndhA (0.56)
ndhH (0.54)
rps15 (0.67)
ycf1 (0.63)
trnR
rrn5
rrn4.5
rrn23
trnA
trnI
rrn16
trnV
trnN
trnL
ndhB
rps7 (0.59)
ycf2 (0.47)
trnH
rps3 (0.54)
rpl22 (0.5)
rps19 (0.74)
rpl2
rpl23 (0.63)
trnI
rpl16
rpl14 (0.5)
rps8 (0.27)
infA (0.5)
rpl36 (0.5)
rps11 (0.42)
rpoA (0.57)
petD (0.51)
petB (0.5)
psbH (0.6)
psbT (0.43)
psbN (0.82)
psbB (0.54)
clpP
rps12
rpl20 (0.8)
psaJ (0.53)
rpl33 (0.67)
rps18 (0.53)
trnR
trnG
atpA (0.48)
atpF (0.51)
atpH (0.5)
atpI (0.48)
rps2 (0.6)
rpoC2 (0.5)
rpoC1 (0.5)
rpoB (0.54)
psbM (0.49)
trnD
trnY
trnE
trnS
trnfM
rps14 (0.54)
psaB (0.26)
psaA (0.44)
ycf3 (0.56)
rps4 (0.54)
trnT
ndhJ (0.49)
ndhK (0.54)
ndhC (0.48)
trnV
atpE (0.53)
atpB (0.5)
