# Supplementary material for: Comparative Analysis of the Chloroplast Genomes of Cypripedium: Assessing the Roles of SSRs and TRs in the Non-Coding Regions of LSC in Shaping Chloroplast Genome Size
Source: Int J Mol Sci. 2025 Apr 14;26(8):3691. doi: 10.3390/ijms26083691 (PMC12027508; doi:10.3390/ijms26083691)
Supplement: Supplementary file 1 [file ijms-26-03691-s001.zip › C. lichiangense.pdf]

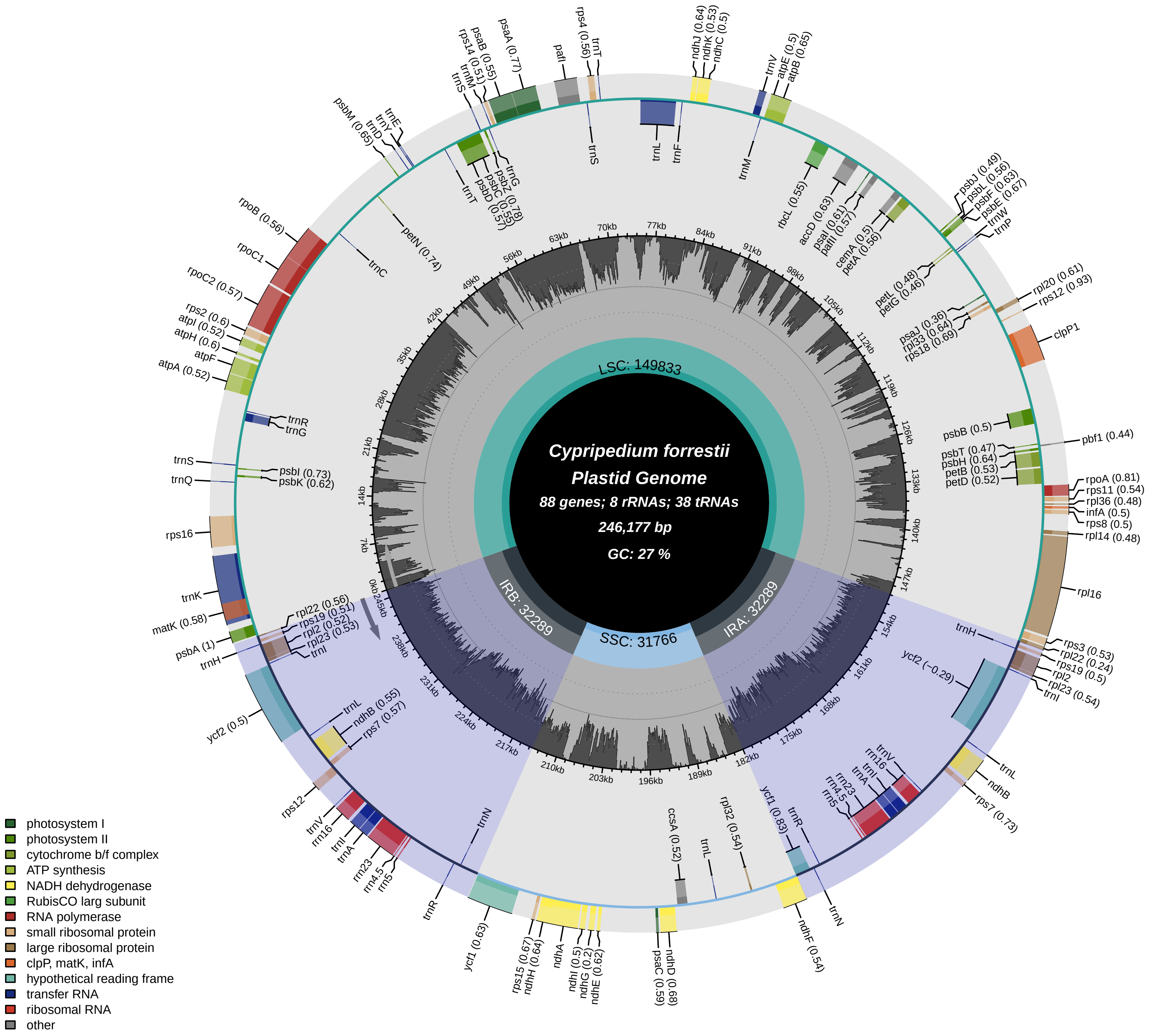
Cypripedium forrestii
Plastid Genome
88 genes; 8 rRNAs; 38 tRNAs
246,177 bp
GC: 27 %
LSC: 149833
IRB: 32289
IRA: 32289
SSC: 31766
0kb
7kb
14kb
21kb
28kb
35kb
42kb
49kb
56kb
63kb
70kb
77kb
84kb
91kb
98kb
105kb
112kb
119kb
126kb
133kb
140kb
147kb
154kb
161kb
168kb
175kb
182kb
189kb
196kb
203kb
210kb
217kb
224kb
231kb
238kb
245kb
psaA (0.77)
psaB (0.55)
rps14 (0.51)
trnfM
trnS
pafI
rps4 (0.56)
trnT
ndhJ (0.64)
ndhK (0.53)
ndhC (0.5)
trnV
atpE (0.5)
atpB (0.65)
psbM (0.65)
trnD
trnY
trnE
trnT
psbD (0.57)
psbC (0.55)
psbZ (0.78)
trnG
trnS
trnL
trnF
trnM
rbcL (0.55)
accD (0.63)
psaI (0.61)
pafII (0.57)
cemA (0.5)
petA (0.56)
psbJ (0.49)
psbL (0.56)
psbF (0.63)
psbE (0.67)
trnW
trnP
petL (0.48)
petG (0.46)
psaJ (0.36)
rpl33 (0.64)
rps18 (0.69)
rpl20 (0.61)
rps12 (0.93)
clpP1
psbB (0.5)
pbf1 (0.44)
psbT (0.47)
psbH (0.64)
petB (0.53)
petD (0.52)
rpoA (0.81)
rps11 (0.54)
rpl36 (0.48)
infA (0.5)
rps8 (0.5)
rpl14 (0.48)
rpl16
trnH
rps3 (0.53)
rpl22 (0.24)
rps19 (0.5)
rpl2
rpl23 (0.54)
trnI
ycf2 (−0.29)
trnL
ndhB
rps7 (0.73)
trnV
rrn16
trnI
trnA
rrn23
rrn4.5
rrn5
trnR
trnN
ycf1 (0.83)
ndhF (0.54)
rpl32 (0.54)
trnL
ccsA (0.52)
ndhD (0.68)
psaC (0.59)
ndhE (0.62)
ndhG (0.2)
ndhI (0.5)
ndhA
ndhH (0.64)
rps15 (0.67)
ycf1 (0.63)
trnR
trnN
rrn5
rrn4.5
rrn23
trnA
trnI
rrn16
trnV
rps12
rps7 (0.57)
ndhB (0.55)
trnL
ycf2 (0.5)
trnH
psbA (1)
matK (0.58)
trnK
rpl22 (0.56)
rps19 (0.51)
rpl2 (0.52)
rpl23 (0.53)
trnI
rps16
trnQ
trnS
psbI (0.73)
psbK (0.62)
trnR
trnG
atpA (0.52)
atpF
atpH (0.6)
atpI (0.52)
rps2 (0.6)
rpoC2 (0.57)
rpoC1
rpoB (0.56)
trnC
petN (0.74)
photosystem I
photosystem II
cytochrome b/f complex
ATP synthesis
NADH dehydrogenase
RubisCO larg subunit
RNA polymerase
small ribosomal protein
large ribosomal protein
clpP, matK, infA
hypothetical reading frame
transfer RNA
ribosomal RNA
other
