# Supplementary material for: Comparative Analysis of the Chloroplast Genomes of Cypripedium: Assessing the Roles of SSRs and TRs in the Non-Coding Regions of LSC in Shaping Chloroplast Genome Size
Source: Int J Mol Sci. 2025 Apr 14;26(8):3691. doi: 10.3390/ijms26083691 (PMC12027508; doi:10.3390/ijms26083691)
Supplement: Supplementary file 1 [file ijms-26-03691-s001.zip › C. micranthum.pdf]

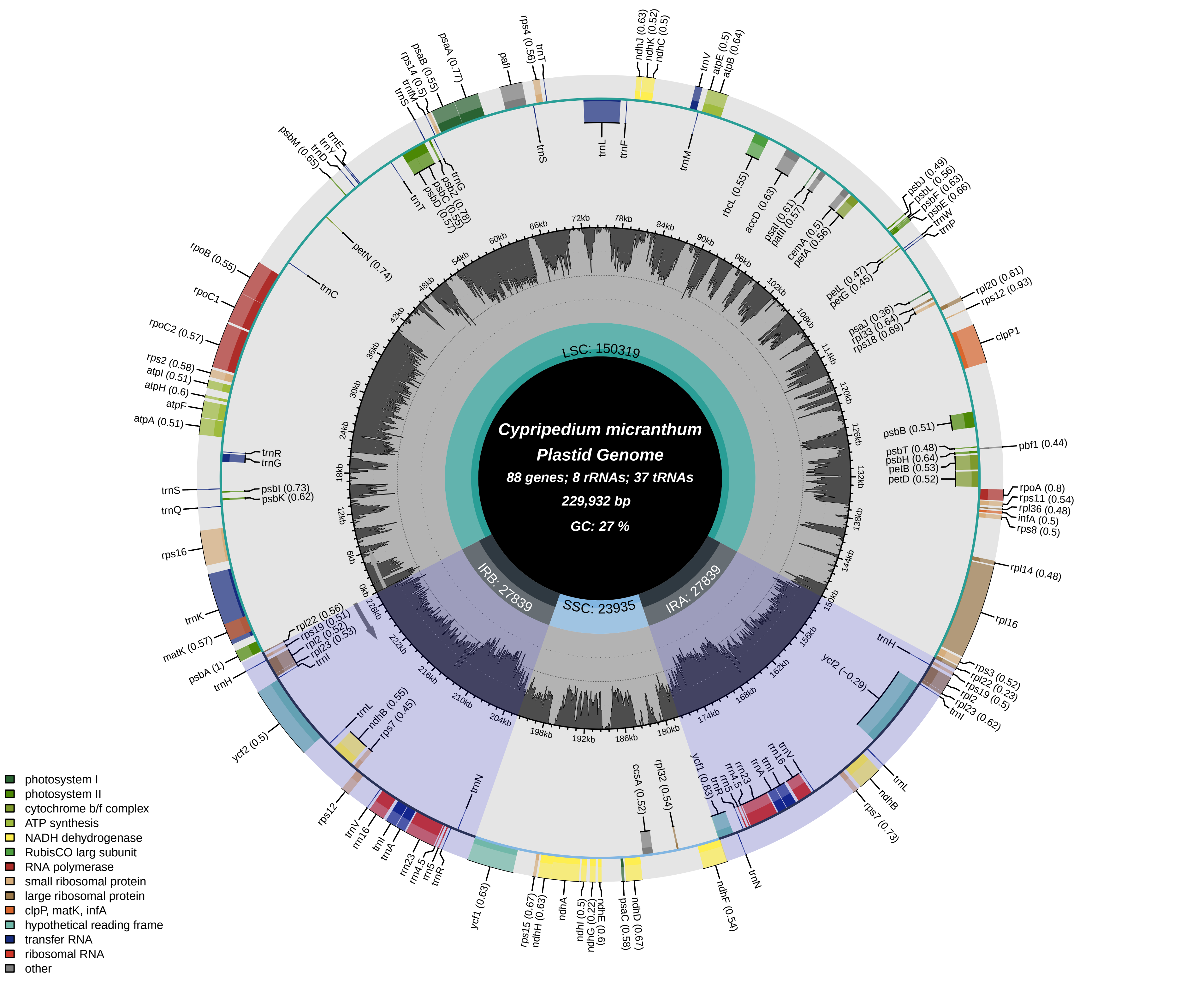
Cypripedium micranthum
Plastid Genome
88 genes; 8 rRNAs; 37 tRNAs
229,932 bp
GC: 27 %
LSC: 150319
IRB: 27839
SSC: 23935
IRA: 27839
psaA (0.77)
psaB (0.55)
rps14 (0.5)
trnfM
trnS
pafI
rps4 (0.56)
trnT
ndhJ (0.63)
ndhK (0.52)
ndhC (0.5)
trnV
atpE (0.5)
atpB (0.64)
trnS
trnL
trnF
trnM
rbcL (0.55)
accD (0.63)
psaI (0.61)
pafII (0.57)
cemA (0.5)
petA (0.56)
psbJ (0.49)
psbL (0.56)
psbF (0.63)
psbE (0.66)
trnW
trnP
petL (0.47)
petG (0.45)
psaJ (0.36)
rpl33 (0.64)
rps18 (0.69)
rpl20 (0.61)
rps12 (0.93)
clpP1
psbB (0.51)
pbf1 (0.44)
psbT (0.48)
psbH (0.64)
petB (0.53)
petD (0.52)
rpoA (0.8)
rps11 (0.54)
rpl36 (0.48)
infA (0.5)
rps8 (0.5)
rpl14 (0.48)
rpl16
trnH
ycf2 (−0.29)
rps3 (0.52)
rpl22 (0.23)
rps19 (0.5)
rpl2
rpl23 (0.62)
trnI
trnL
ndhB
rps7 (0.73)
trnV
rrn16
trnI
trnA
rrn23
rrn4.5
rrn5
trnR
ycf1 (0.83)
ccsA (0.52)
rpl32 (0.54)
ndhF (0.54)
trnN
ndhD (0.67)
psaC (0.58)
ndhE (0.6)
ndhG (0.22)
ndhI (0.5)
ndhA
ndhH (0.63)
rps15 (0.67)
ycf1 (0.63)
trnN
rrn23
rrn4.5
rrn5
trnR
trnA
trnI
rrn16
trnV
rps12
ycf2 (0.5)
trnL
ndhB (0.55)
rps7 (0.45)
trnH
psbA (1)
matK (0.57)
trnK
rpl22 (0.56)
rps19 (0.51)
rpl2 (0.52)
rpl23 (0.53)
trnI
rps16
trnQ
trnS
psbI (0.73)
psbK (0.62)
trnR
trnG
atpA (0.51)
atpF
atpH (0.6)
atpI (0.51)
rps2 (0.58)
rpoC2 (0.57)
rpoC1
rpoB (0.55)
trnC
petN (0.74)
psbM (0.65)
trnD
trnY
trnE
trnT
psbD (0.57)
psbC (0.55)
psbZ (0.78)
trnG
0kb
6kb
12kb
18kb
24kb
30kb
36kb
42kb
48kb
54kb
60kb
66kb
72kb
78kb
84kb
90kb
96kb
102kb
108kb
114kb
120kb
126kb
132kb
138kb
144kb
150kb
156kb
162kb
168kb
174kb
180kb
186kb
192kb
198kb
204kb
210kb
216kb
222kb
228kb
photosystem I
photosystem II
cytochrome b/f complex
ATP synthesis
NADH dehydrogenase
RubisCO larg subunit
RNA polymerase
small ribosomal protein
large ribosomal protein
clpP, matK, infA
hypothetical reading frame
transfer RNA
ribosomal RNA
other
