# Supplementary material for: Comparative Analysis of the Chloroplast Genomes of Cypripedium: Assessing the Roles of SSRs and TRs in the Non-Coding Regions of LSC in Shaping Chloroplast Genome Size
Source: Int J Mol Sci. 2025 Apr 14;26(8):3691. doi: 10.3390/ijms26083691 (PMC12027508; doi:10.3390/ijms26083691)
Supplement: Supplementary file 1 [file ijms-26-03691-s001.zip › C. plectrochilum.pdf]

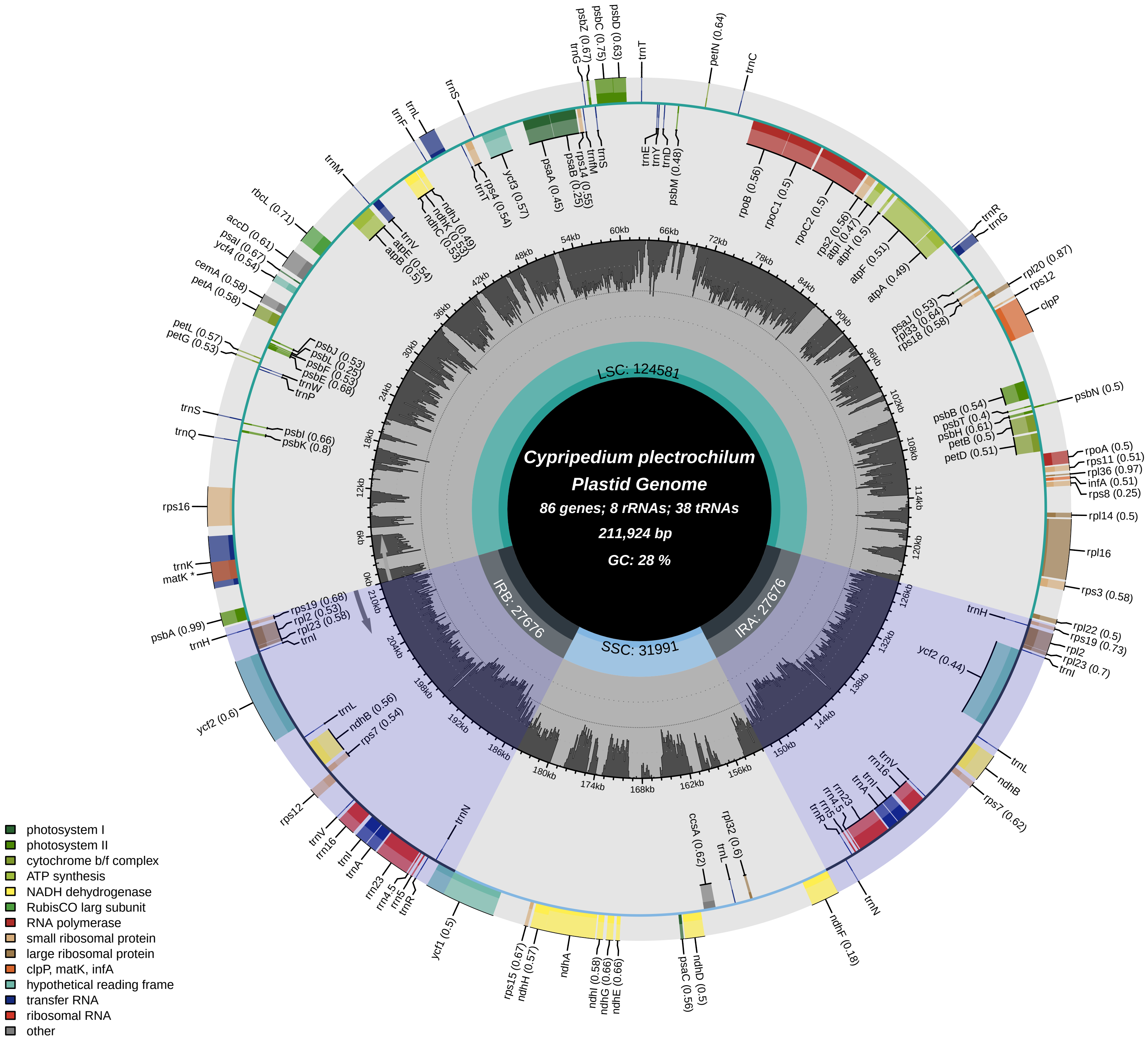
Cypripedium plectrochilum
Plastid Genome
86 genes; 8 rRNAs; 38 tRNAs
211,924 bp
GC: 28 %
LSC: 124581
IRA: 27676
SSC: 31991
IRB: 27676
0kb
6kb
12kb
18kb
24kb
30kb
36kb
42kb
48kb
54kb
60kb
66kb
72kb
78kb
84kb
90kb
96kb
102kb
108kb
114kb
120kb
126kb
132kb
138kb
144kb
150kb
156kb
162kb
168kb
174kb
180kb
186kb
192kb
198kb
204kb
210kb
psbA (0.99)
trnH
trnK
matK *
rps16
trnQ
trnS
psbI (0.66)
psbK (0.8)
petL (0.57)
petG (0.53)
psbJ (0.53)
psbL (0.25)
psbF (0.53)
psbE (0.68)
trnW
trnP
petA (0.58)
cemA (0.58)
ycf4 (0.54)
psaI (0.67)
accD (0.61)
rbcL (0.71)
trnM
atpE (0.54)
atpB (0.5)
trnV
ndhC (0.53)
ndhK (0.53)
ndhJ (0.49)
trnF
trnL
trnT
rps4 (0.54)
trnS
ycf3 (0.57)
psaA (0.45)
psaB (0.25)
rps14 (0.55)
trnfM
trnS
trnG
psbZ (0.67)
psbC (0.75)
psbD (0.63)
trnT
trnE
trnY
trnD
psbM (0.48)
petN (0.64)
trnC
rpoB (0.56)
rpoC1 (0.5)
rpoC2 (0.5)
rps2 (0.56)
atpI (0.47)
atpH (0.5)
atpF (0.51)
atpA (0.49)
trnR
trnG
rpl20 (0.87)
rps12
clpP
psaJ (0.53)
rpl33 (0.64)
rps18 (0.58)
psbN (0.5)
psbB (0.54)
psbT (0.4)
psbH (0.61)
petB (0.5)
petD (0.51)
rpoA (0.5)
rps11 (0.51)
rpl36 (0.97)
infA (0.51)
rps8 (0.25)
rpl14 (0.5)
rpl16
rps3 (0.58)
trnH
rpl22 (0.5)
rps19 (0.73)
rpl2
rpl23 (0.7)
trnI
ycf2 (0.44)
trnL
ndhB
rps7 (0.62)
trnV
rrn16
trnI
trnA
rrn23
rrn4.5
rrn5
trnR
trnN
ndhF (0.18)
rpl32 (0.6)
trnL
ccsA (0.62)
ndhD (0.5)
psaC (0.56)
ndhE (0.66)
ndhG (0.66)
ndhI (0.58)
ndhA
ndhH (0.57)
rps15 (0.67)
ycf1 (0.5)
trnN
trnR
rrn5
rrn4.5
rrn23
trnA
trnI
rrn16
trnV
rps12
rps7 (0.54)
ndhB (0.56)
trnL
ycf2 (0.6)
rps19 (0.68)
rpl2 (0.53)
rpl23 (0.58)
trnI
photosystem I
photosystem II
cytochrome b/f complex
ATP synthesis
NADH dehydrogenase
RubisCO larg subunit
RNA polymerase
small ribosomal protein
large ribosomal protein
clpP, matK, infA
hypothetical reading frame
transfer RNA
ribosomal RNA
other
