# Supplementary material for: Comparative Analysis of the Chloroplast Genomes of Cypripedium: Assessing the Roles of SSRs and TRs in the Non-Coding Regions of LSC in Shaping Chloroplast Genome Size
Source: Int J Mol Sci. 2025 Apr 14;26(8):3691. doi: 10.3390/ijms26083691 (PMC12027508; doi:10.3390/ijms26083691)
Supplement: Supplementary file 1 [file ijms-26-03691-s001.zip › Supplementary Figures and File.pdf]

Supplementary Figures and File

Figure S1 The average chloroplast genome length of the seven sections in *Cypripedium*.

Figure S2 Structural alignments of 18 *Cypripedium* chloroplast genomes based on the Mauve analysis. The red boxes represent inversions in the SSC and IR regions.

Figure S3 Visualization of alignment of 18 *Cypripedium* species chloroplast genome sequences. VISTA-based identity plots showed sequence identity of seven chloroplast genomes with *C. debile* as a reference.

Figure S4 the Ka/Ks ratios for 74 genes across 17 species using *C. debile* as a reference.

Figure S5 Number of different SSRs types in 18 *Cypripedium* species.

Figure S6 the Codon usage and bias in the chloroplast genomes of 18 *Cypripedium* species using CodonW software.

Supplementary data File1 Chloroplast genome coding sequences (CDS) of 33 orchid species.
