# Supplementary figures and images for: Comparative Analysis of the Chloroplast Genomes of Cypripedium: Assessing the Roles of SSRs and TRs in the Non-Coding Regions of LSC in Shaping Chloroplast Genome Size
Source: Int J Mol Sci. 2025 Apr 14;26(8):3691. doi: 10.3390/ijms26083691 (PMC12027508; doi:10.3390/ijms26083691)

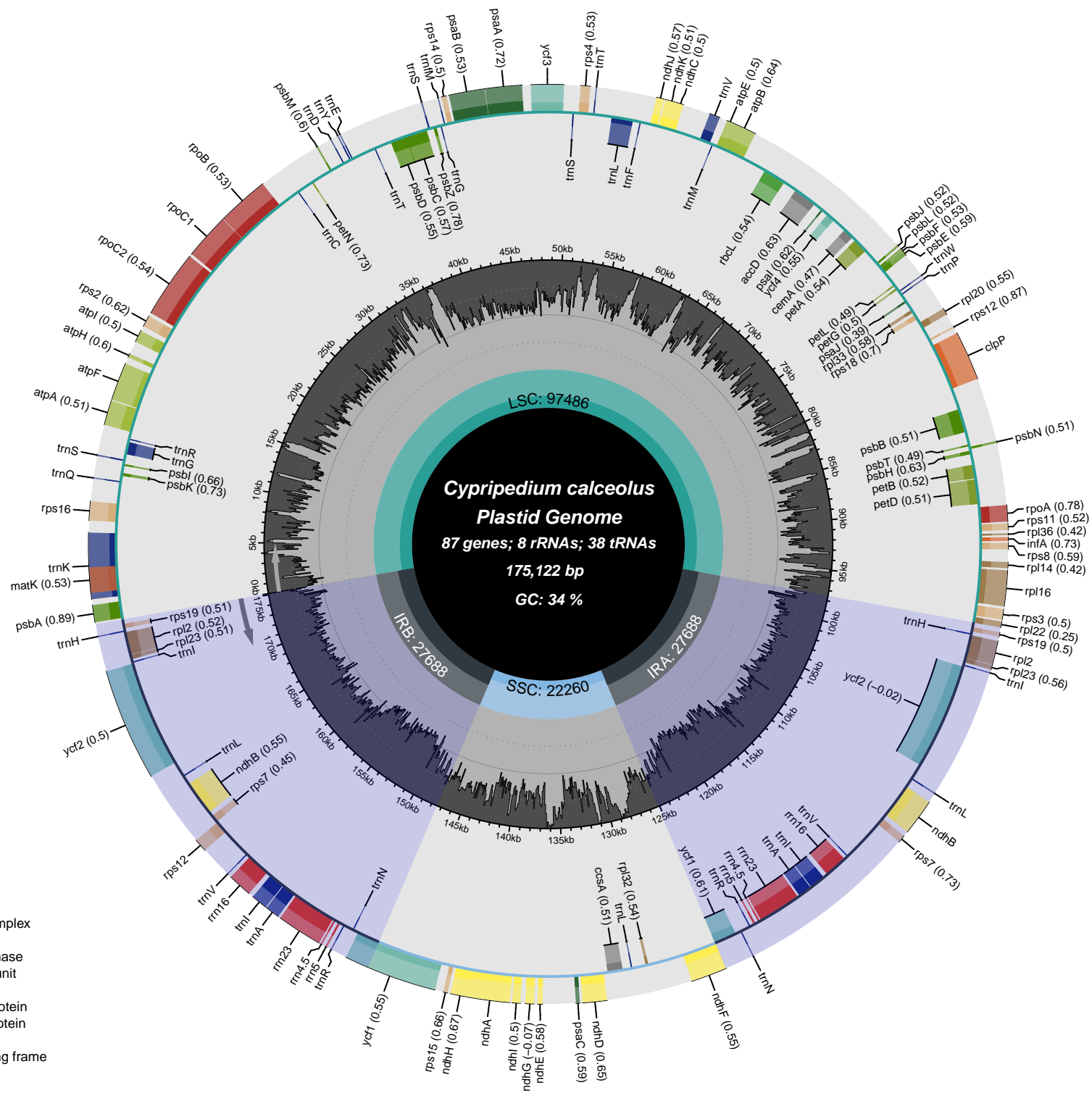

Supplement: Supplementary file 1 [file ijms-26-03691-s001.zip › C. calceolus.pdf]

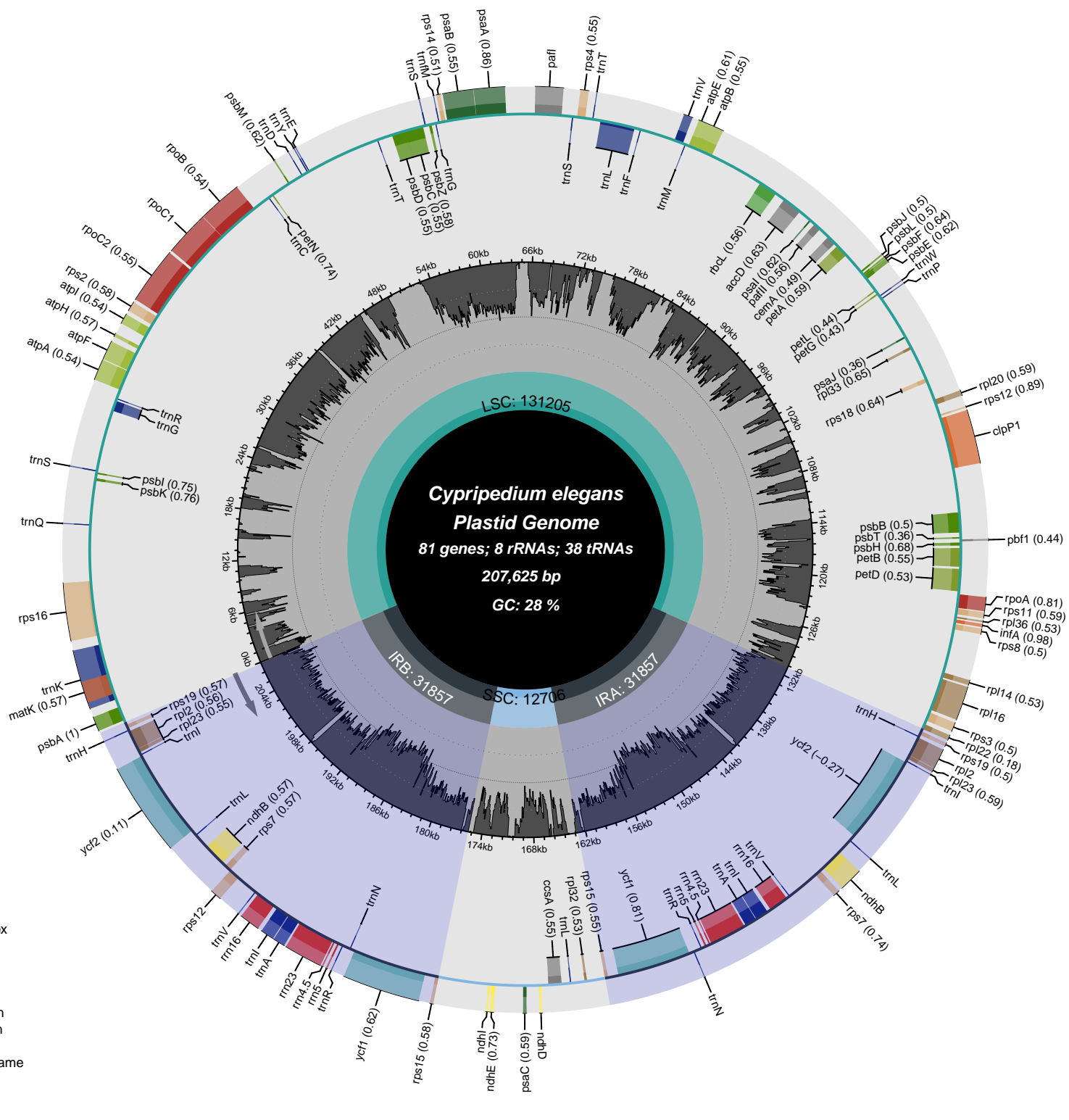

Supplement: Supplementary file 1 [file ijms-26-03691-s001.zip › C. elegans.pdf]

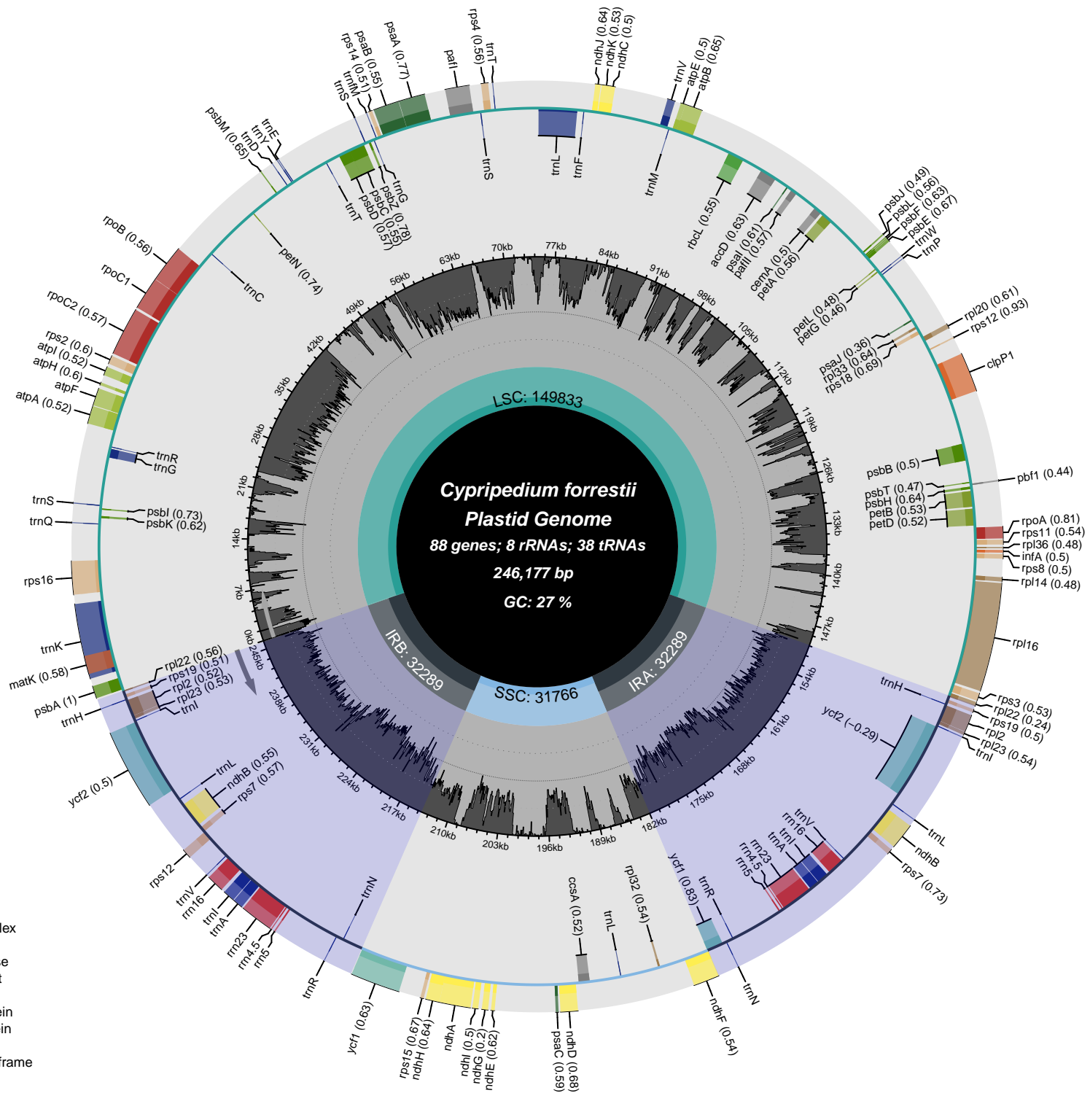

Supplement: Supplementary file 1 [file ijms-26-03691-s001.zip › C. fargesii.pdf]

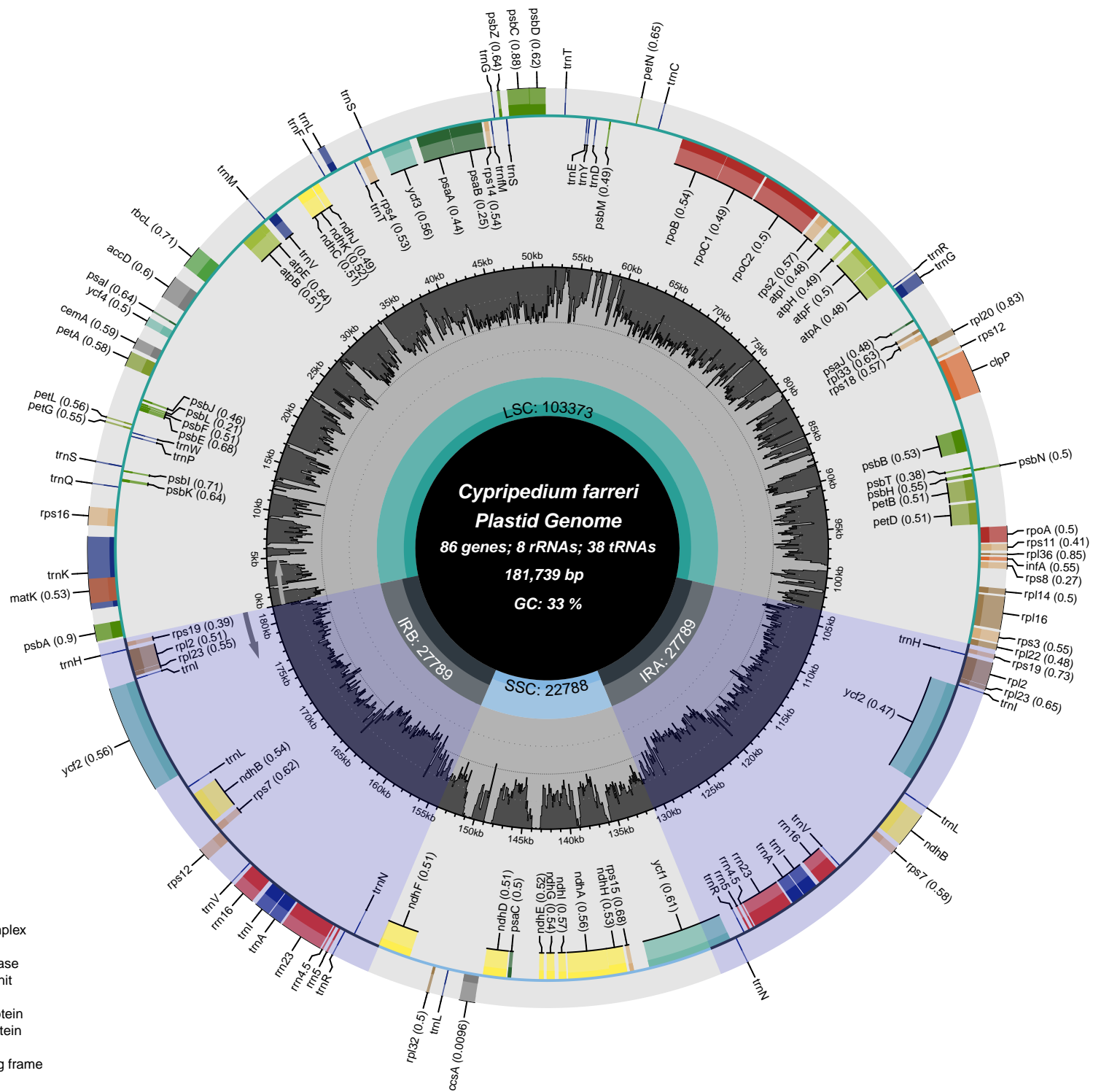

Supplement: Supplementary file 1 [file ijms-26-03691-s001.zip › C. farreri.pdf]

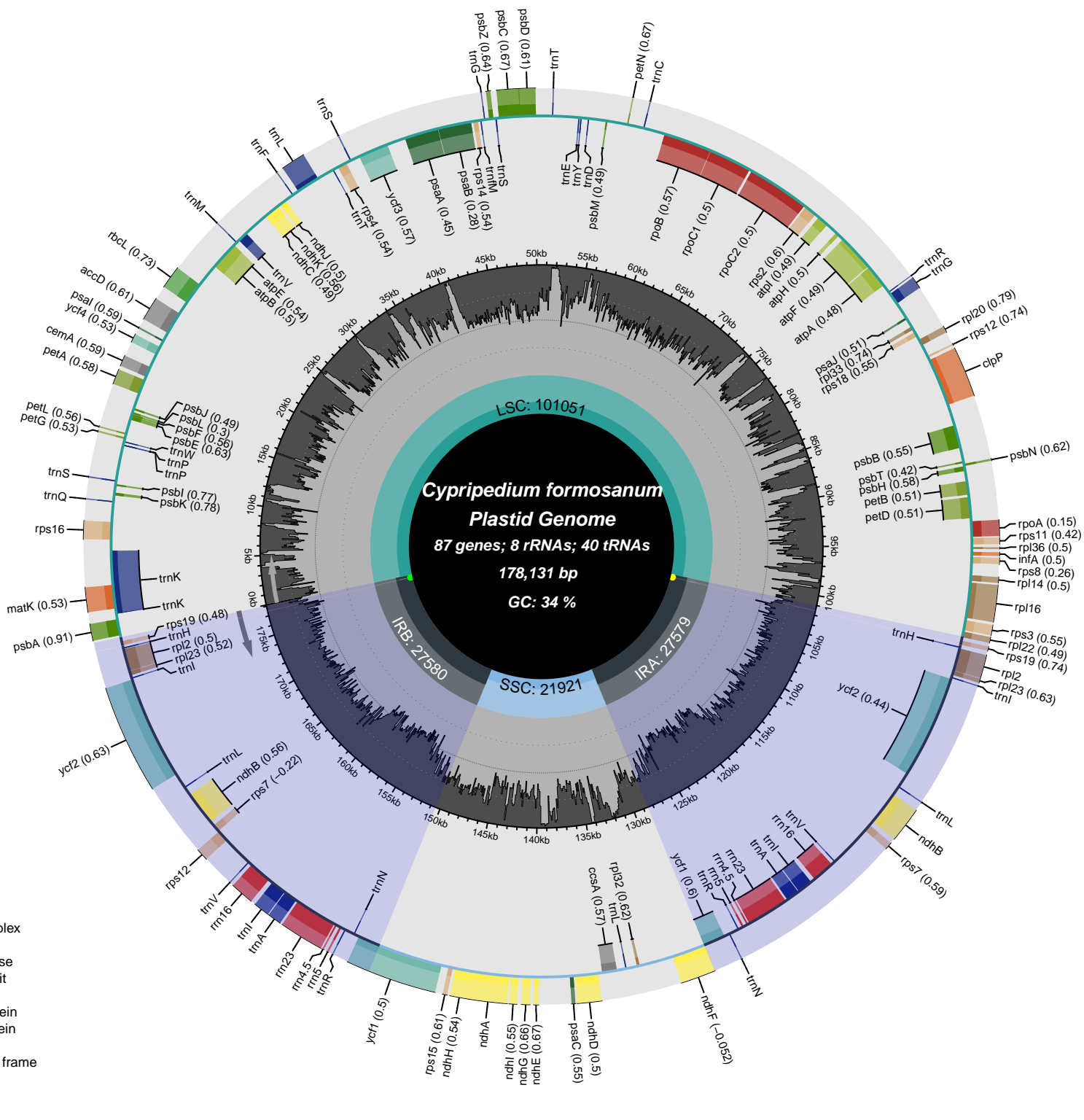

Supplement: Supplementary file 1 [file ijms-26-03691-s001.zip › C. formosanum.pdf]

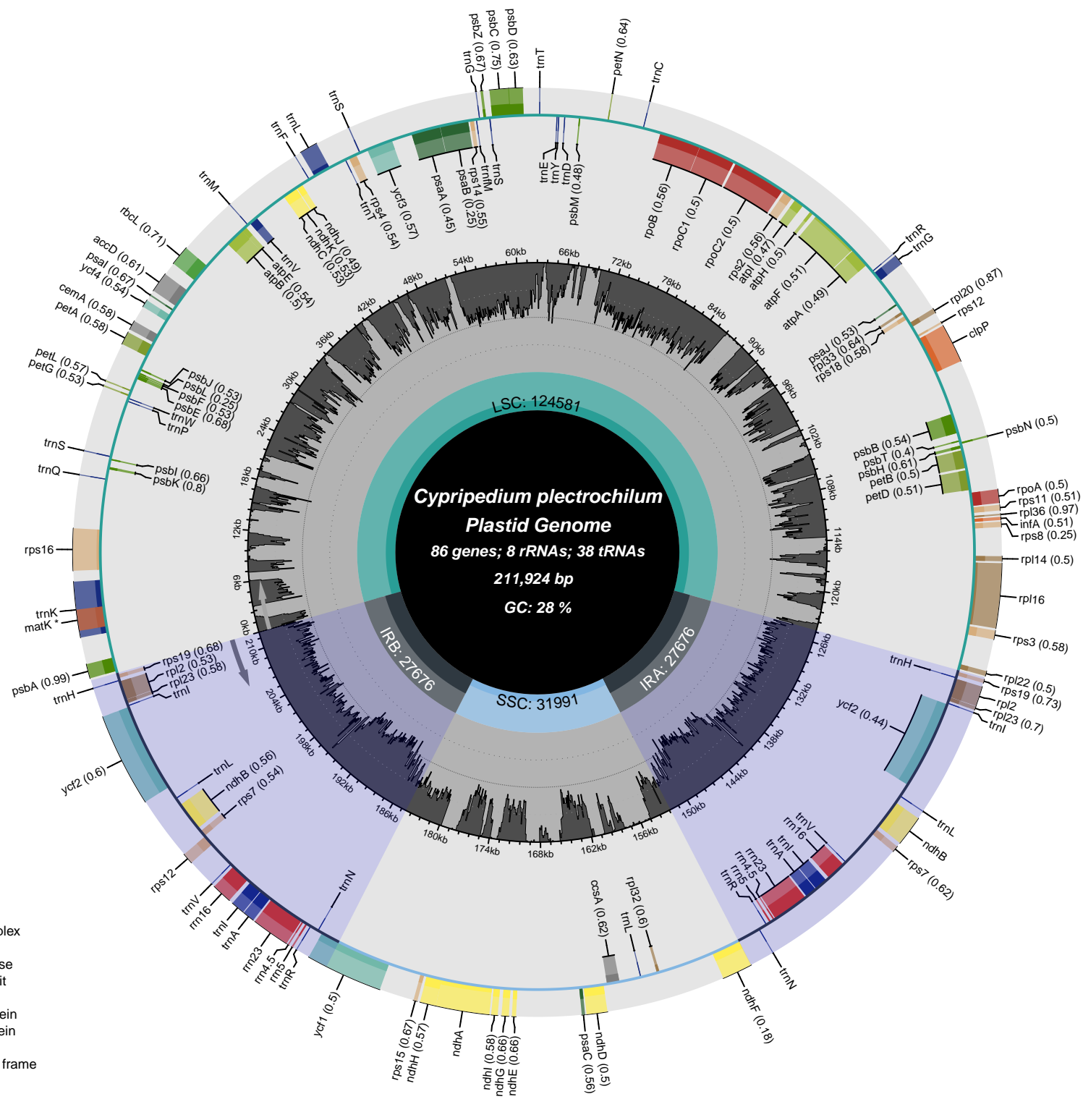

Supplement: Supplementary file 1 [file ijms-26-03691-s001.zip › C. guttatum.pdf]

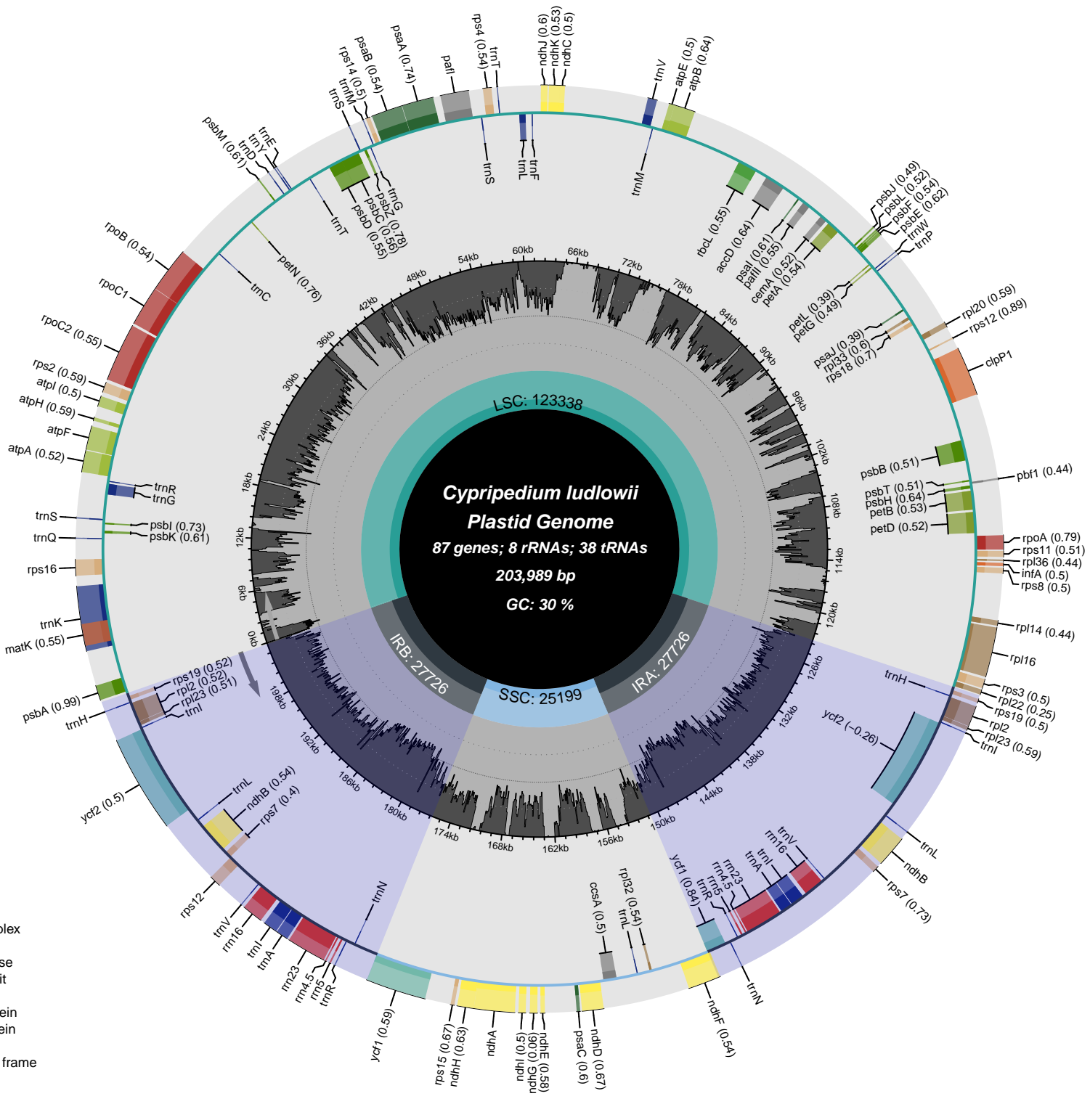

Supplement: Supplementary file 1 [file ijms-26-03691-s001.zip › C. ludlowii.pdf]

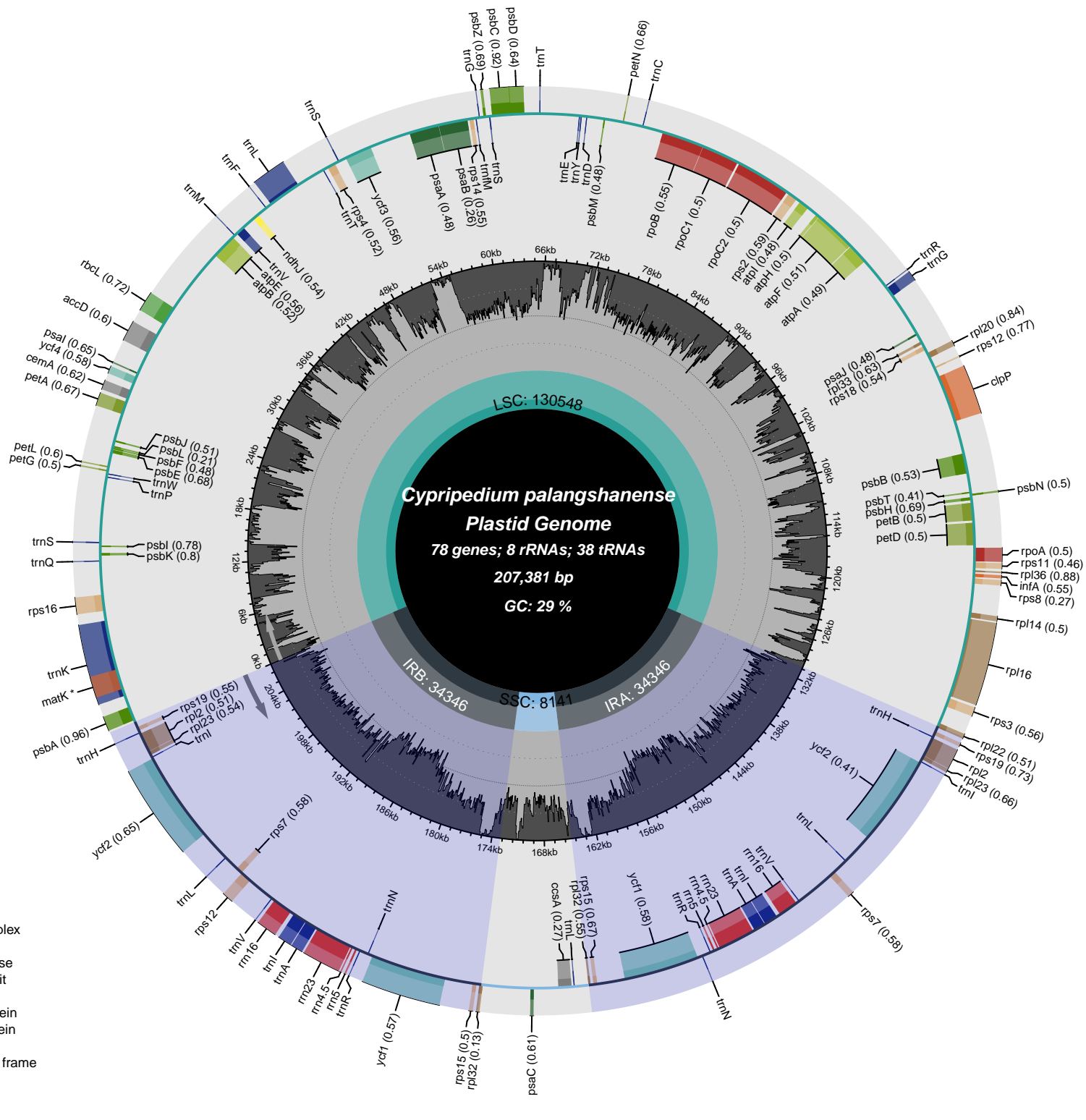

Supplement: Supplementary file 1 [file ijms-26-03691-s001.zip › C. palangshanense.pdf]

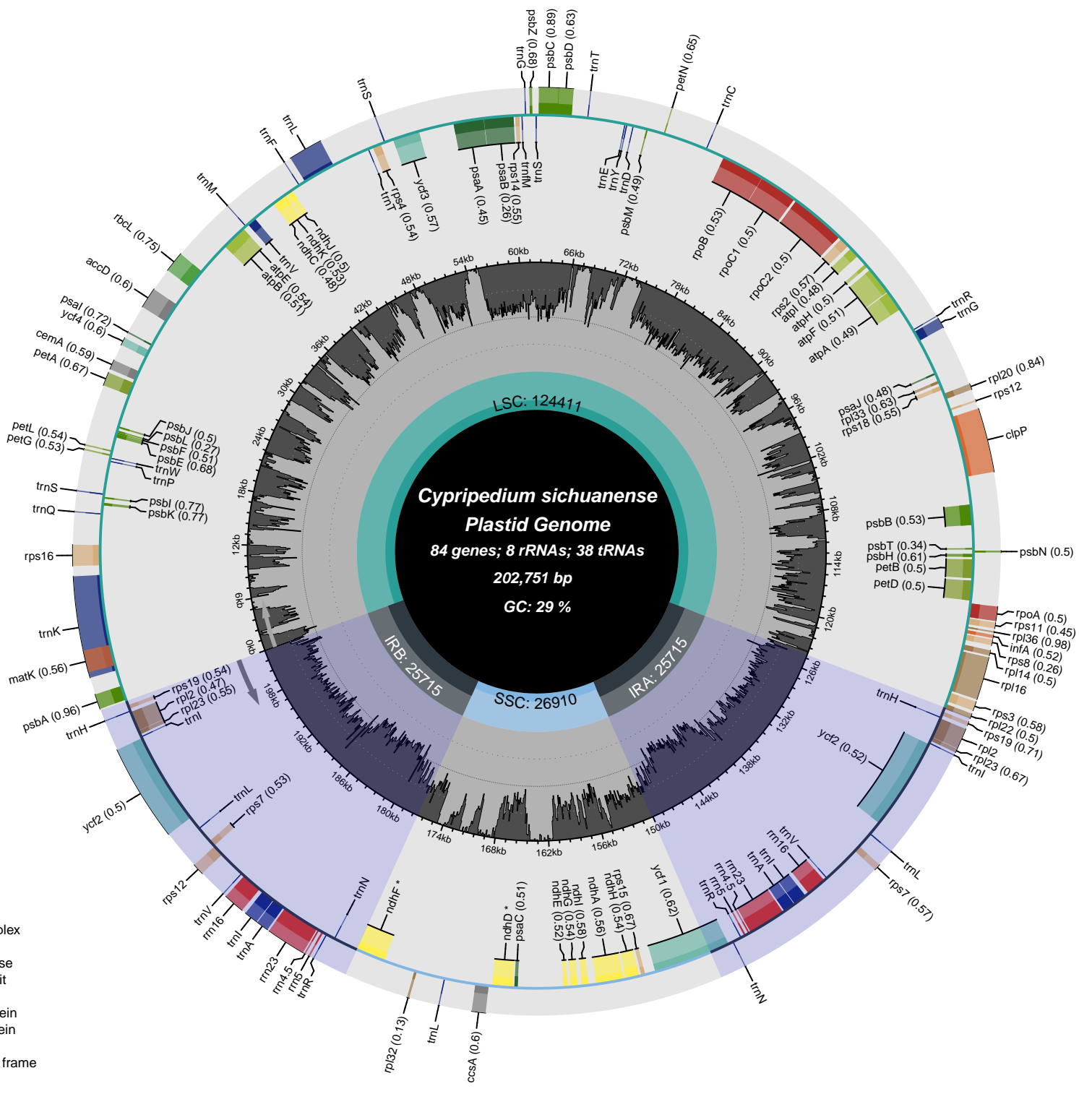

Supplement: Supplementary file 1 [file ijms-26-03691-s001.zip › C. sichuanense.pdf]

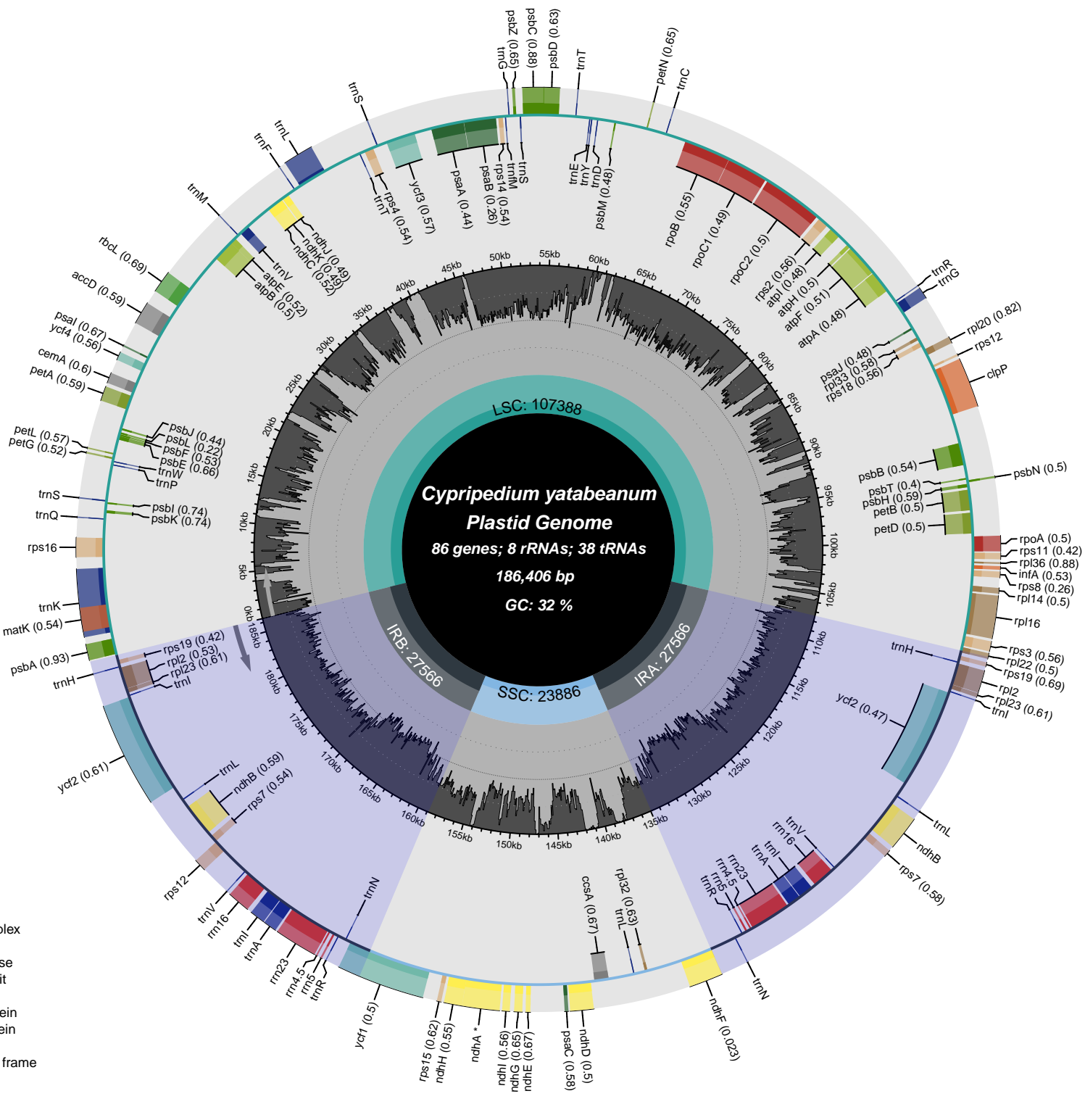

Supplement: Supplementary file 1 [file ijms-26-03691-s001.zip › C. subtropicum.pdf]

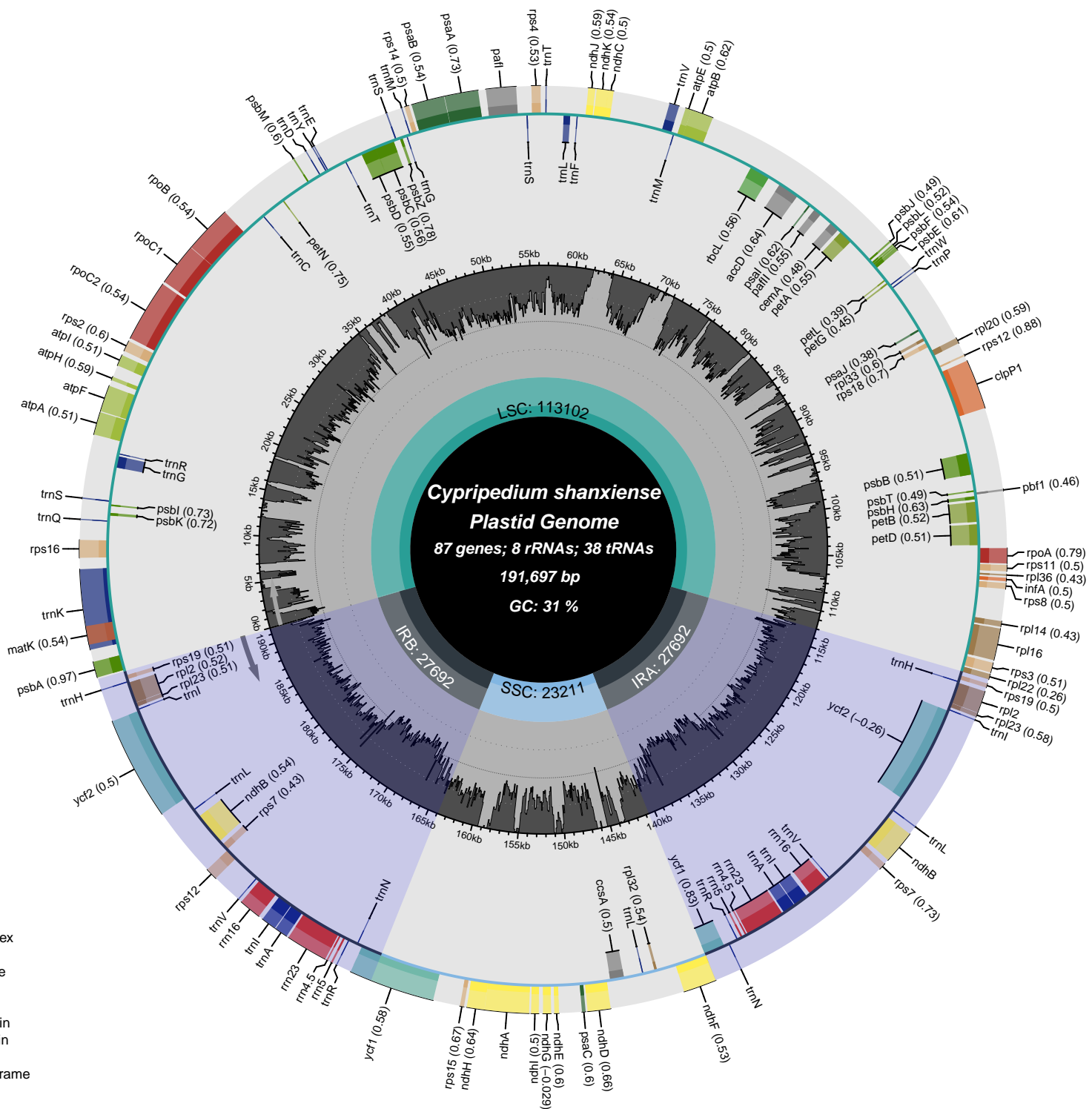

Supplement: Supplementary file 1 [file ijms-26-03691-s001.zip › C. tibeticum.pdf]

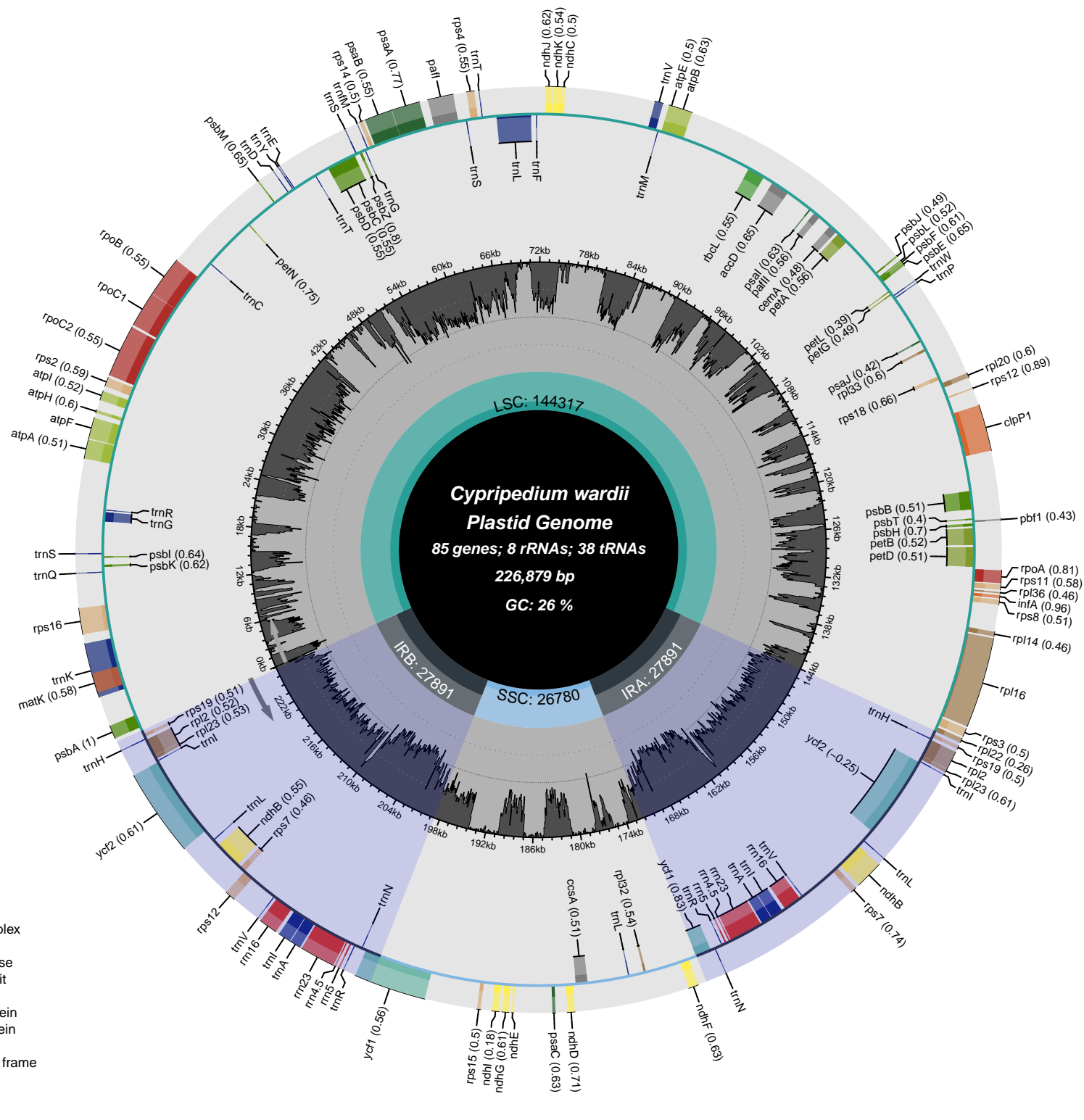

Supplement: Supplementary file 1 [file ijms-26-03691-s001.zip › C. wardii.pdf]

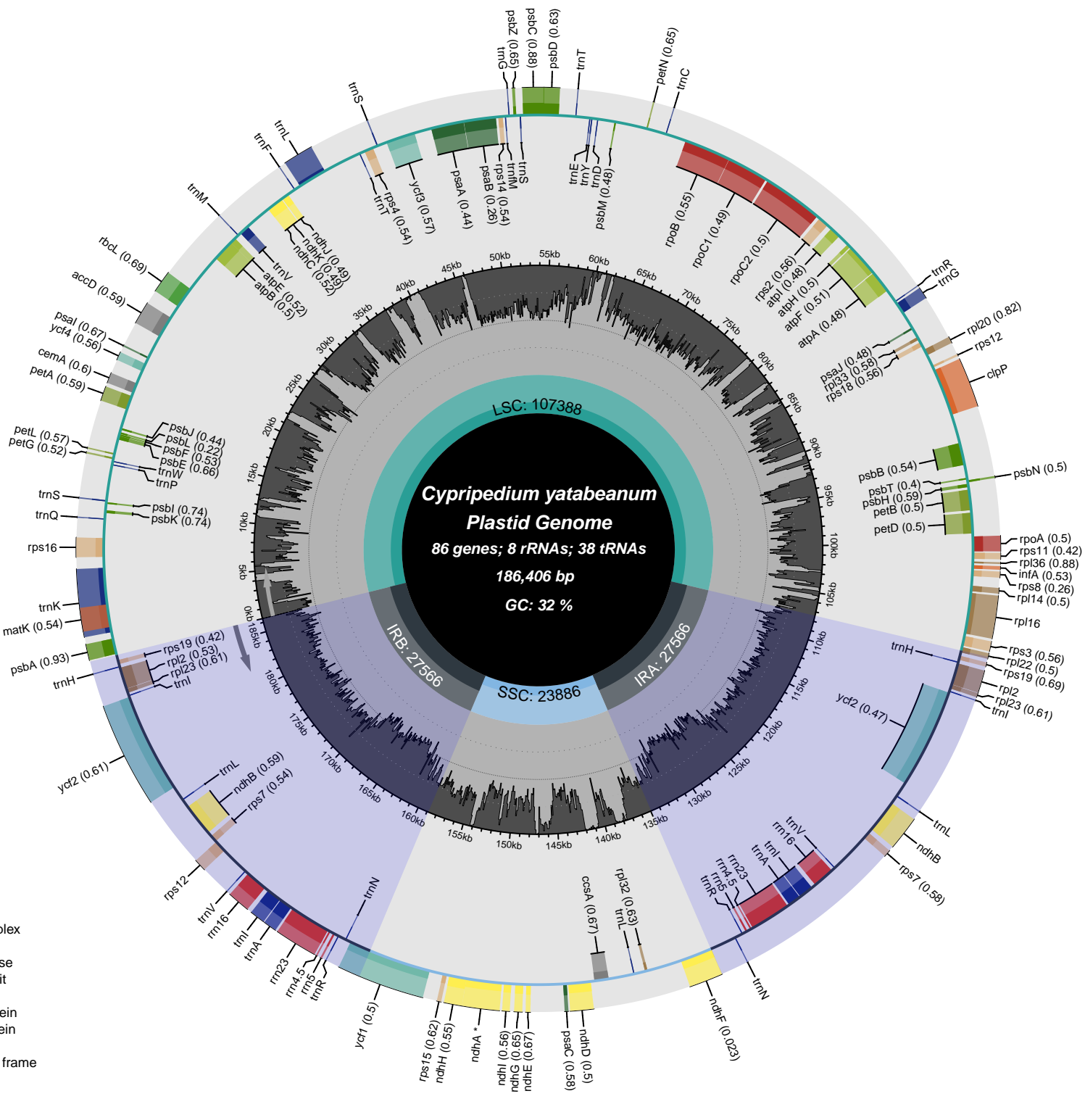

Supplement: Supplementary file 1 [file ijms-26-03691-s001.zip › C. yatabeanum.pdf]

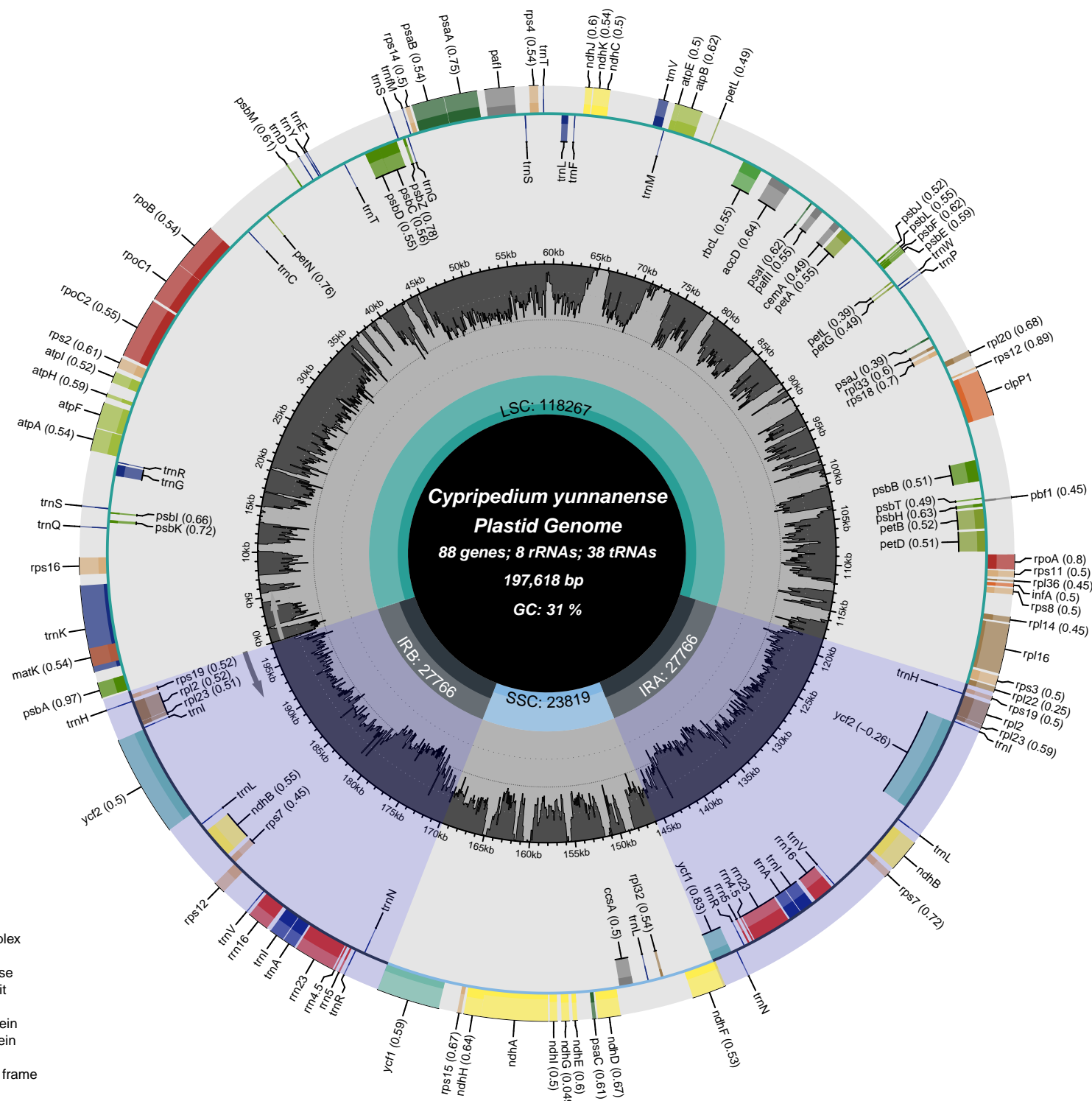

Supplement: Supplementary file 1 [file ijms-26-03691-s001.zip › C. yunnanense.pdf]

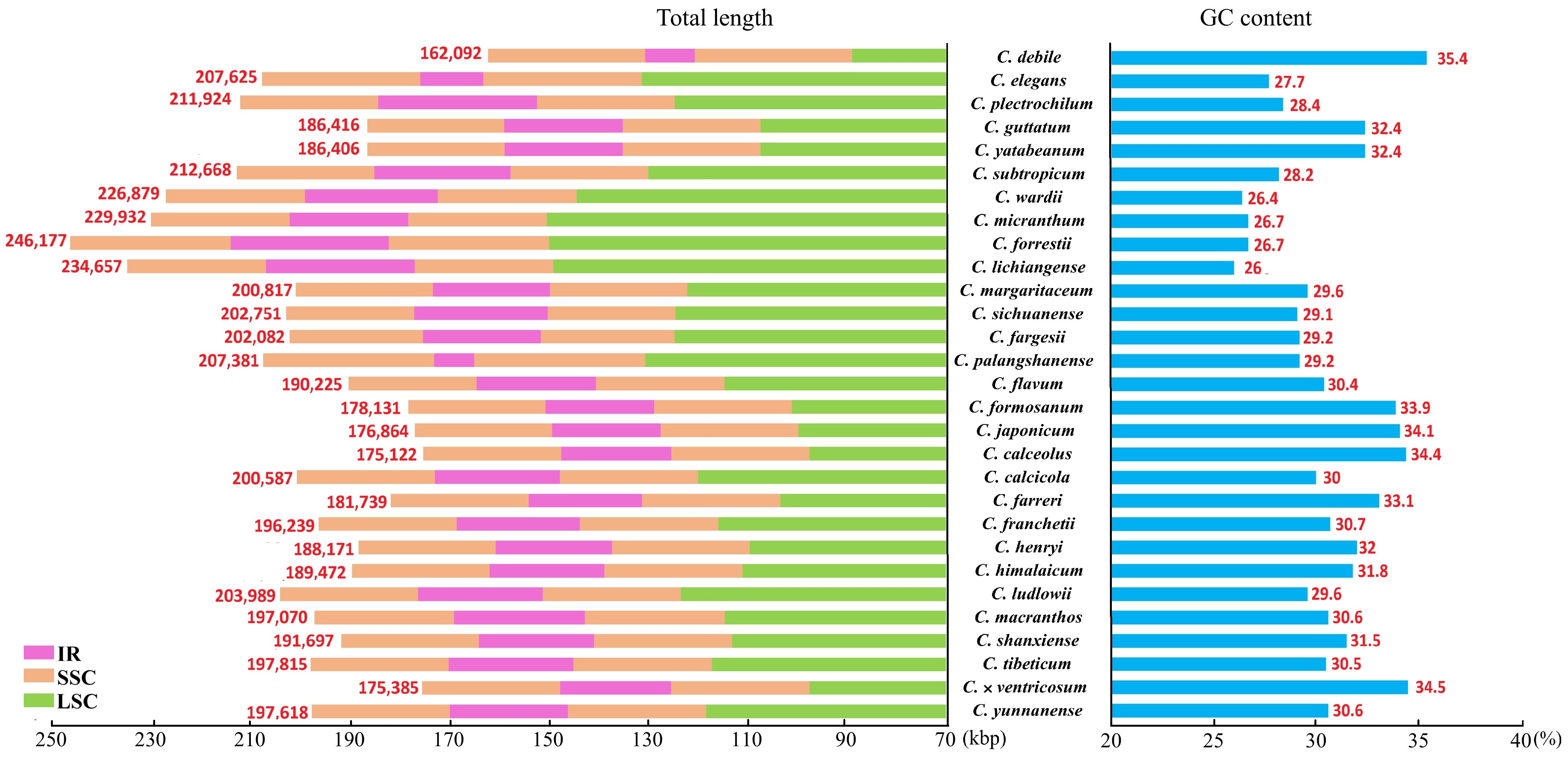

Supplement: Supplementary file 1 [file ijms-26-03691-s001.zip › Figure S1.jpg]

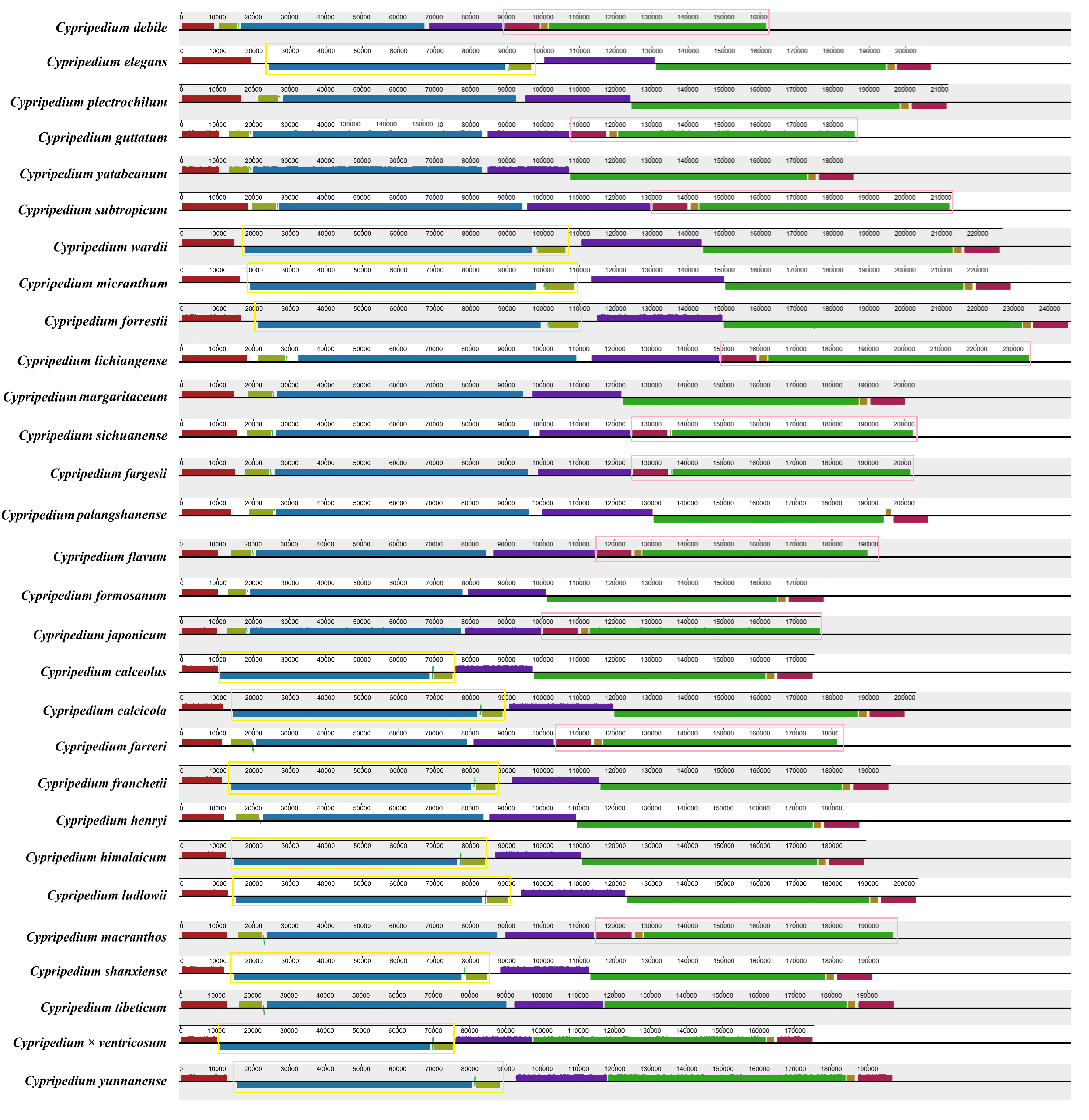

Supplement: Supplementary file 1 [file ijms-26-03691-s001.zip › Figure S3.tif]

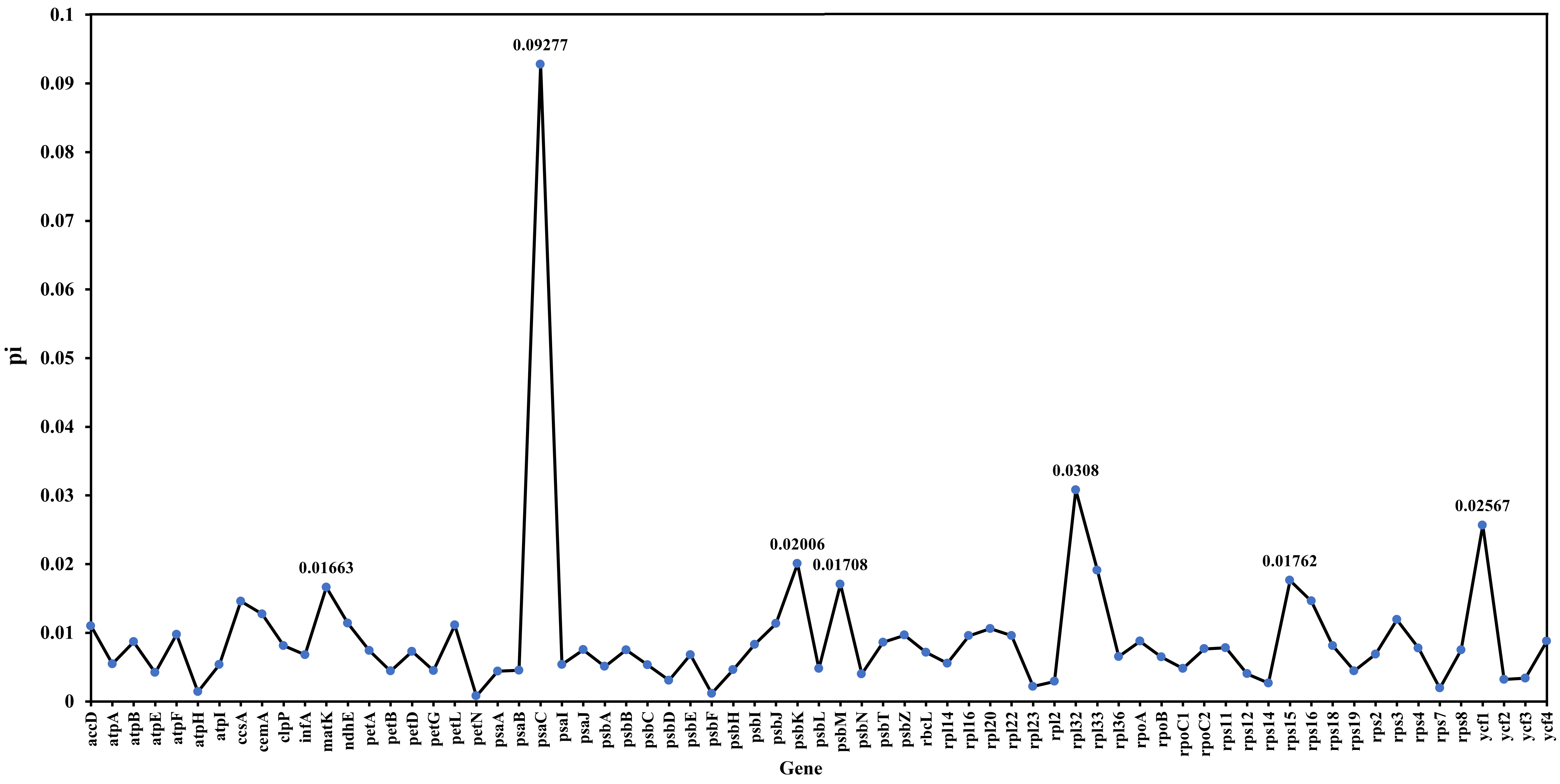

Supplement: Supplementary file 1 [file ijms-26-03691-s001.zip › Figure S5.png]

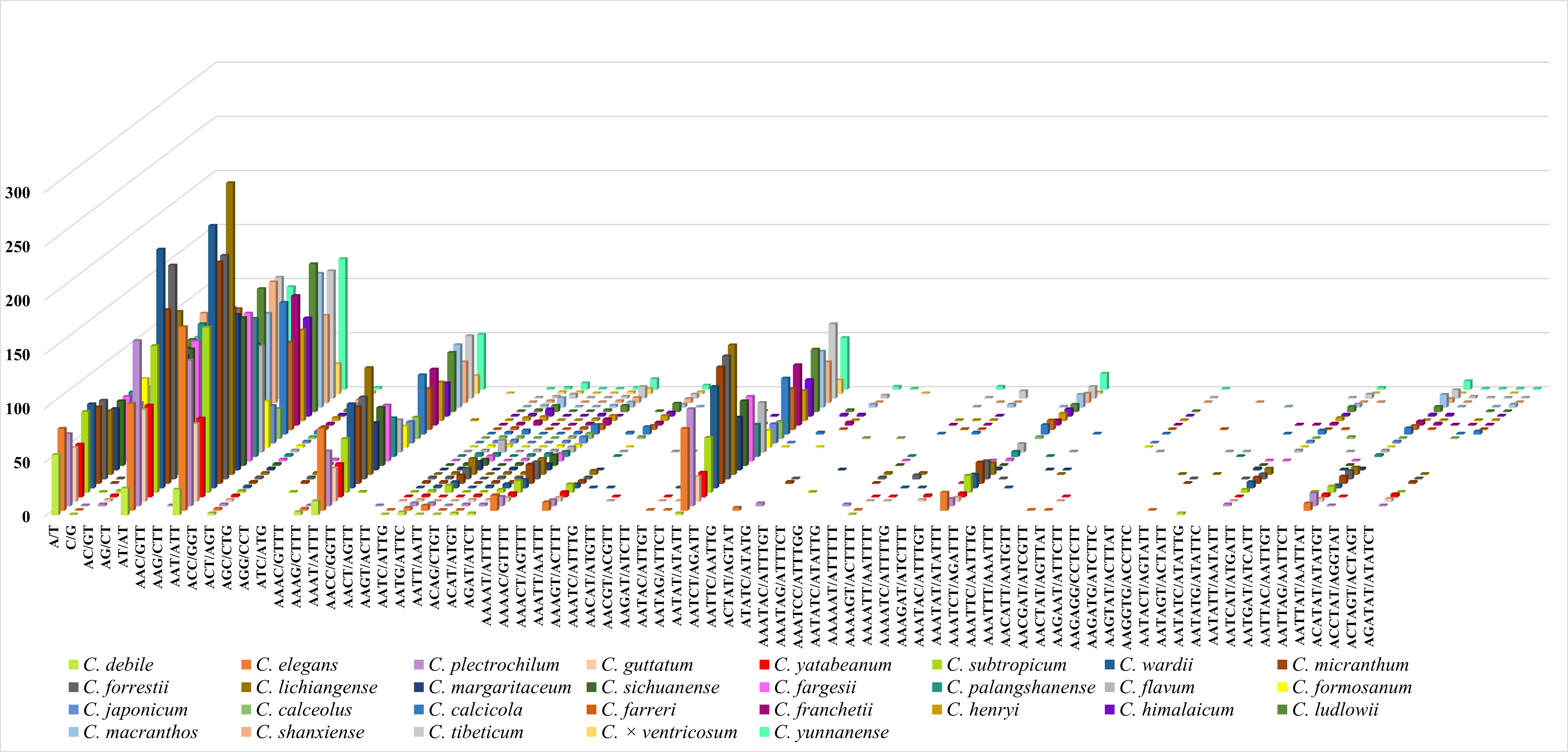

Supplement: Supplementary file 1 [file ijms-26-03691-s001.zip › Figure S6.png]
