# Supplementary material for: Comparative Analysis of the Chloroplast Genomes of Cypripedium: Assessing the Roles of SSRs and TRs in the Non-Coding Regions of LSC in Shaping Chloroplast Genome Size
Source: Int J Mol Sci. 2025 Apr 14;26(8):3691. doi: 10.3390/ijms26083691 (PMC12027508; doi:10.3390/ijms26083691)
Supplement: Supplementary file 1 [file C. calcicola.pdf › C. calcicola.pdf]

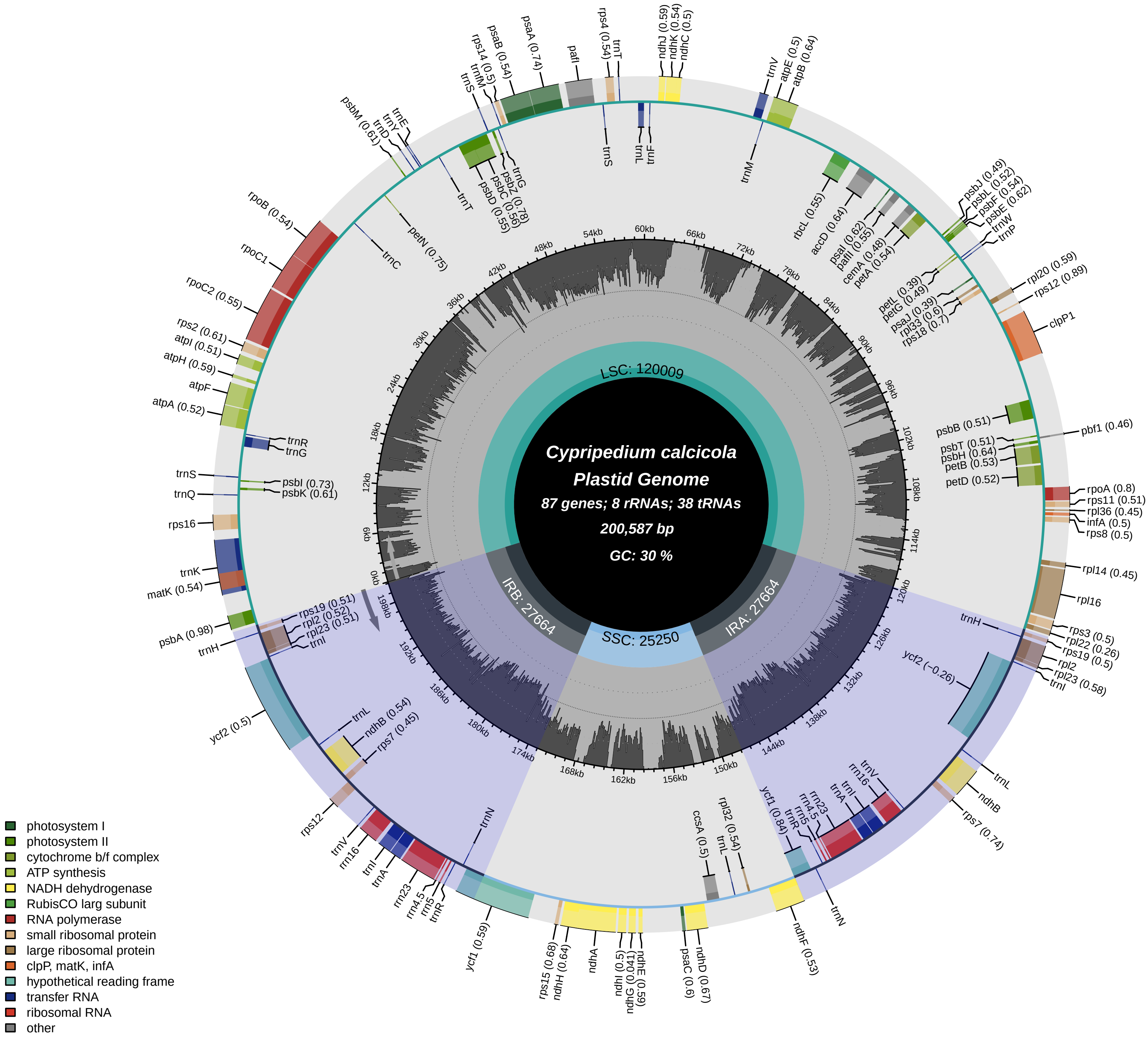

Cypripedium calcicola
Plastid Genome
87 genes; 8 rRNAs; 38 tRNAs
200,587 bp
GC: 30 %
LSC: 120009
IRA: 27664
SSC: 25250
IRB: 27664
0kb
6kb
12kb
18kb
24kb
30kb
36kb
42kb
48kb
54kb
60kb
66kb
72kb
78kb
84kb
90kb
96kb
102kb
108kb
114kb
120kb
126kb
132kb
138kb
144kb
150kb
156kb
162kb
168kb
174kb
180kb
186kb
192kb
198kb
psbA (0.98)
trnH
matK (0.54)
trnK
rps16
trnQ
trnS
psbI (0.73)
psbK (0.61)
trnR
trnG
atpA (0.52)
atpF
atpH (0.59)
atpI (0.51)
rps2 (0.61)
rpoC2 (0.55)
rpoC1
rpoB (0.54)
trnC
petN (0.75)
psbM (0.61)
trnD
trnY
trnE
trnT
psbD (0.55)
psbC (0.56)
psbZ (0.78)
trnG
trnS
trnfM
rps14 (0.5)
psaB (0.54)
psaA (0.74)
pafI
rps4 (0.54)
trnT
trnS
trnL
trnF
ndhJ (0.59)
ndhK (0.54)
ndhC (0.5)
trnM
trnV
atpE (0.5)
atpB (0.64)
rbcL (0.55)
accD (0.64)
psaI (0.62)
pafII (0.55)
cemA (0.48)
petA (0.54)
psbJ (0.49)
psbL (0.52)
psbF (0.54)
psbE (0.62)
trnW
trnP
petL (0.39)
petG (0.49)
psaJ (0.39)
rpl33 (0.6)
rps18 (0.7)
rpl20 (0.59)
rps12 (0.89)
clpP1
psbB (0.51)
pbf1 (0.46)
psbT (0.51)
psbH (0.64)
petB (0.53)
petD (0.52)
rpoA (0.8)
rps11 (0.51)
rpl36 (0.45)
infA (0.5)
rps8 (0.5)
rpl14 (0.45)
rpl16
trnH
rps3 (0.5)
rpl22 (0.26)
rps19 (0.5)
rpl2
rpl23 (0.58)
trnI
ycf2 (−0.26)
trnL
ndhB
rps7 (0.74)
trnV
rrn16
trnI
trnA
rrn23
rrn4.5
rrn5
trnR
ycf1 (0.84)
trnN
ndhF (0.53)
rpl32 (0.54)
trnL
ccsA (0.5)
ndhD (0.67)
psaC (0.6)
ndhE (0.59)
ndhG (0.041)
ndhI (0.5)
ndhA
ndhH (0.64)
rps15 (0.68)
ycf1 (0.59)
trnN
trnR
rrn5
rrn4.5
rrn23
trnA
trnI
rrn16
trnV
rps12
rps7 (0.45)
ndhB (0.54)
trnL
ycf2 (0.5)
rps19 (0.51)
rpl2 (0.52)
rpl23 (0.51)
trnI
trnH
photosystem I
photosystem II
cytochrome b/f complex
ATP synthesis
NADH dehydrogenase
RubisCO larg subunit
RNA polymerase
small ribosomal protein
large ribosomal protein
clpP, matK, infA
hypothetical reading frame
transfer RNA
ribosomal RNA
other
