# Supplementary material for: Comparative Analysis of the Chloroplast Genomes of Cypripedium: Assessing the Roles of SSRs and TRs in the Non-Coding Regions of LSC in Shaping Chloroplast Genome Size
Source: Int J Mol Sci. 2025 Apr 14;26(8):3691. doi: 10.3390/ijms26083691 (PMC12027508; doi:10.3390/ijms26083691)
Supplement: Supplementary file 1 [file C. debile.pdf › C. debile.pdf]

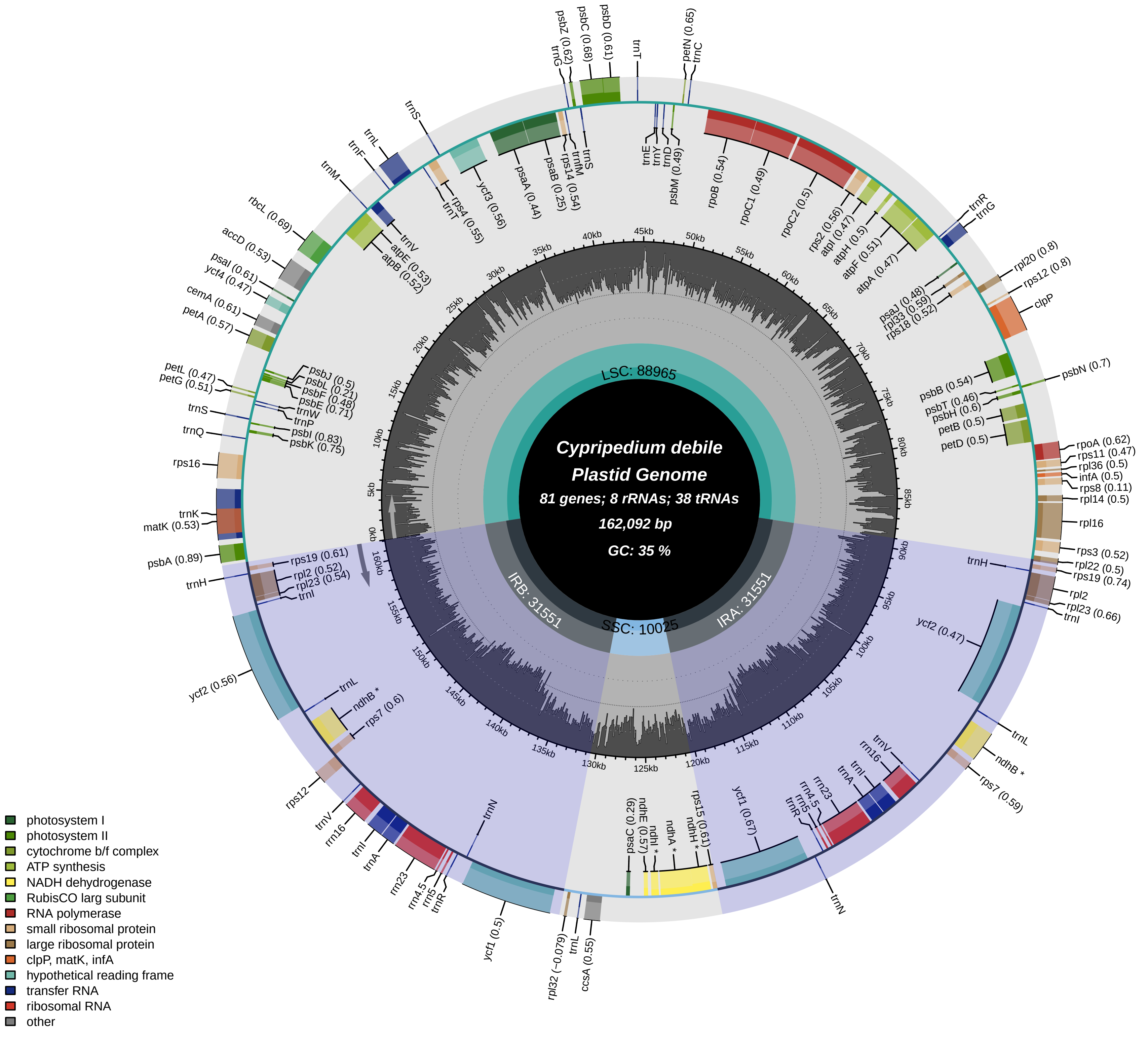

Cypripedium debile
Plastid Genome
81 genes; 8 rRNAs; 38 tRNAs
162,092 bp
GC: 35 %
LSC: 88965
IRB: 31551
IRA: 31551
SSC: 10025
psbZ (0.62)
trnG
psbC (0.68)
psbD (0.61)
trnT
petN (0.65)
trnC
trnE
trnY
trnD
psbM (0.49)
rpoB (0.54)
rpoC1 (0.49)
rpoC2 (0.5)
rps2 (0.56)
atpI (0.47)
atpH (0.5)
atpF (0.51)
atpA (0.47)
trnR
trnG
rpl20 (0.8)
rps12 (0.8)
clpP
psaJ (0.48)
rpl33 (0.59)
rps18 (0.52)
psbN (0.7)
psbB (0.54)
psbT (0.46)
psbH (0.6)
petB (0.5)
petD (0.5)
rpoA (0.62)
rps11 (0.47)
rpl36 (0.5)
infA (0.5)
rps8 (0.11)
rpl14 (0.5)
rpl16
rps3 (0.52)
trnH
rpl22 (0.5)
rps19 (0.74)
rpl2
rpl23 (0.66)
trnI
ycf2 (0.47)
trnL
ndhB *
rps7 (0.59)
trnV
rrn16
trnI
trnA
rrn23
rrn4.5
rrn5
trnR
trnN
ycf1 (0.67)
rps15 (0.61)
ndhH *
ndhA *
ndhI *
ndhE (0.57)
psaC (0.29)
ccsA (0.55)
trnL
rpl32 (~0.079)
ycf1 (0.5)
trnN
trnR
rrn5
rrn4.5
rrn23
trnA
trnI
rrn16
trnV
rps12
rps7 (0.6)
ndhB *
trnL
ycf2 (0.56)
trnH
trnI
rpl23 (0.54)
rpl2 (0.52)
rps19 (0.61)
psbA (0.89)
matK (0.53)
trnK
rps16
trnQ
trnS
psbK (0.75)
psbI (0.83)
trnP
trnW
psbE (0.71)
psbF (0.48)
psbL (0.21)
psbJ (0.5)
petG (0.51)
petL (0.47)
petA (0.57)
cemA (0.61)
ycf4 (0.47)
psaI (0.61)
accD (0.53)
rbcL (0.69)
trnM
atpB (0.52)
atpE (0.53)
trnV
trnF
trnL
trnT
rps4 (0.55)
trnS
ycf3 (0.56)
psaA (0.44)
psaB (0.25)
rps14 (0.54)
trnfM
trnS
0kb
5kb
10kb
15kb
20kb
25kb
30kb
35kb
40kb
45kb
50kb
55kb
60kb
65kb
70kb
75kb
80kb
85kb
90kb
95kb
100kb
105kb
110kb
115kb
120kb
125kb
130kb
135kb
140kb
145kb
150kb
155kb
160kb
photosystem I
photosystem II
cytochrome b/f complex
ATP synthesis
NADH dehydrogenase
RubisCO larg subunit
RNA polymerase
small ribosomal protein
large ribosomal protein
clpP, matK, infA
hypothetical reading frame
transfer RNA
ribosomal RNA
other
