# Supplementary material for: Comparative Analysis of the Chloroplast Genomes of Cypripedium: Assessing the Roles of SSRs and TRs in the Non-Coding Regions of LSC in Shaping Chloroplast Genome Size
Source: Int J Mol Sci. 2025 Apr 14;26(8):3691. doi: 10.3390/ijms26083691 (PMC12027508; doi:10.3390/ijms26083691)
Supplement: Supplementary file 1 [file C. henryi.pdf › C. henryi.pdf]

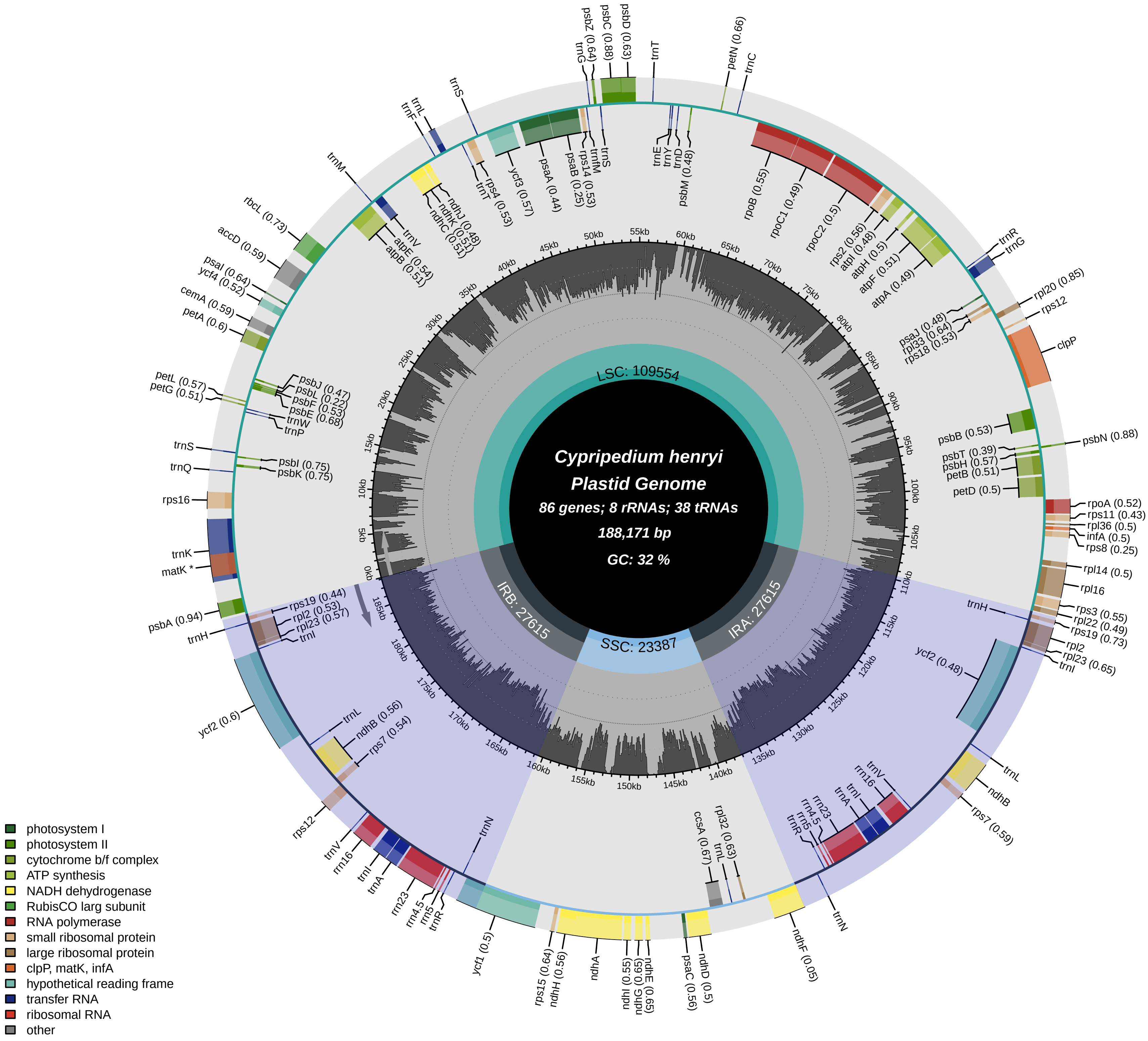
Cypripedium henryi
Plastid Genome
86 genes; 8 rRNAs; 38 tRNAs
188,171 bp
GC: 32 %
LSC: 109554
IRA: 27615
SSC: 23387
IRB: 27615
0kb
5kb
10kb
15kb
20kb
25kb
30kb
35kb
40kb
45kb
50kb
55kb
60kb
65kb
70kb
75kb
80kb
85kb
90kb
95kb
100kb
105kb
110kb
115kb
120kb
125kb
130kb
135kb
140kb
145kb
150kb
155kb
160kb
165kb
170kb
175kb
180kb
185kb
trnG
psbZ (0.64)
psbC (0.88)
psbD (0.63)
trnT
petN (0.66)
trnC
trnS
trnL
trnF
trnM
rbcL (0.73)
accD (0.59)
psaI (0.64)
ycf4 (0.52)
cemA (0.59)
petA (0.6)
petL (0.57)
petG (0.51)
psbJ (0.47)
psbL (0.22)
psbF (0.53)
psbE (0.68)
trnW
trnP
trnS
psbI (0.75)
trnQ
psbK (0.75)
rps16
trnK
matK *
psbA (0.94)
trnH
rps19 (0.44)
rpl2 (0.53)
rpl23 (0.57)
trnI
ycf2 (0.6)
trnL
ndhB (0.56)
rps7 (0.54)
rps12
trnV
rrn16
trnI
trnA
rrn23
rrn4.5
rrn5
trnR
trnN
ycf1 (0.5)
rps15 (0.64)
ndhH (0.56)
ndhA
ndhI (0.55)
ndhG (0.6)
ndhE (0.65)
psaC (0.56)
ndhD (0.5)
ccsA (0.67)
trnL
rpl32 (0.63)
ndhF (0.05)
trnN
trnR
rrn5
rrn4.5
rrn23
trnA
trnI
rrn16
trnV
rps7 (0.59)
ndhB
trnL
ycf2 (0.48)
trnI
rpl23 (0.65)
rpl2
rps19 (0.73)
rpl22 (0.49)
rps3 (0.55)
trnH
rpl16
rpl14 (0.5)
rps8 (0.25)
infA (0.5)
rpl36 (0.5)
rps11 (0.43)
rpoA (0.52)
petD (0.5)
petB (0.51)
psbH (0.57)
psbT (0.39)
psbN (0.88)
psbB (0.53)
clpP
rps12
rpl20 (0.85)
psaJ (0.48)
rpl33 (0.64)
rps18 (0.53)
trnR
trnG
atpA (0.49)
atpF (0.51)
atpH (0.5)
atpI (0.48)
rps2 (0.56)
rpoC2 (0.5)
rpoC1 (0.49)
rpoB (0.55)
psbM (0.48)
trnD
trnY
trnE
trnS
trnfM
rps14 (0.53)
psaB (0.25)
psaA (0.44)
ycf3 (0.57)
rps4 (0.53)
trnT
ndhJ (0.48)
ndhK (0.51)
ndhC (0.51)
trnV
atpE (0.54)
atpB (0.51)
photosystem I
photosystem II
cytochrome b/f complex
ATP synthesis
NADH dehydrogenase
RubisCO larg subunit
RNA polymerase
small ribosomal protein
large ribosomal protein
clpP, matK, infA
hypothetical reading frame
transfer RNA
ribosomal RNA
other
