# Supplementary material for: Comparative Analysis of the Chloroplast Genomes of Cypripedium: Assessing the Roles of SSRs and TRs in the Non-Coding Regions of LSC in Shaping Chloroplast Genome Size
Source: Int J Mol Sci. 2025 Apr 14;26(8):3691. doi: 10.3390/ijms26083691 (PMC12027508; doi:10.3390/ijms26083691)
Supplement: Supplementary file 1 [file C. himalaicum.pdf › C. himalaicum.pdf]

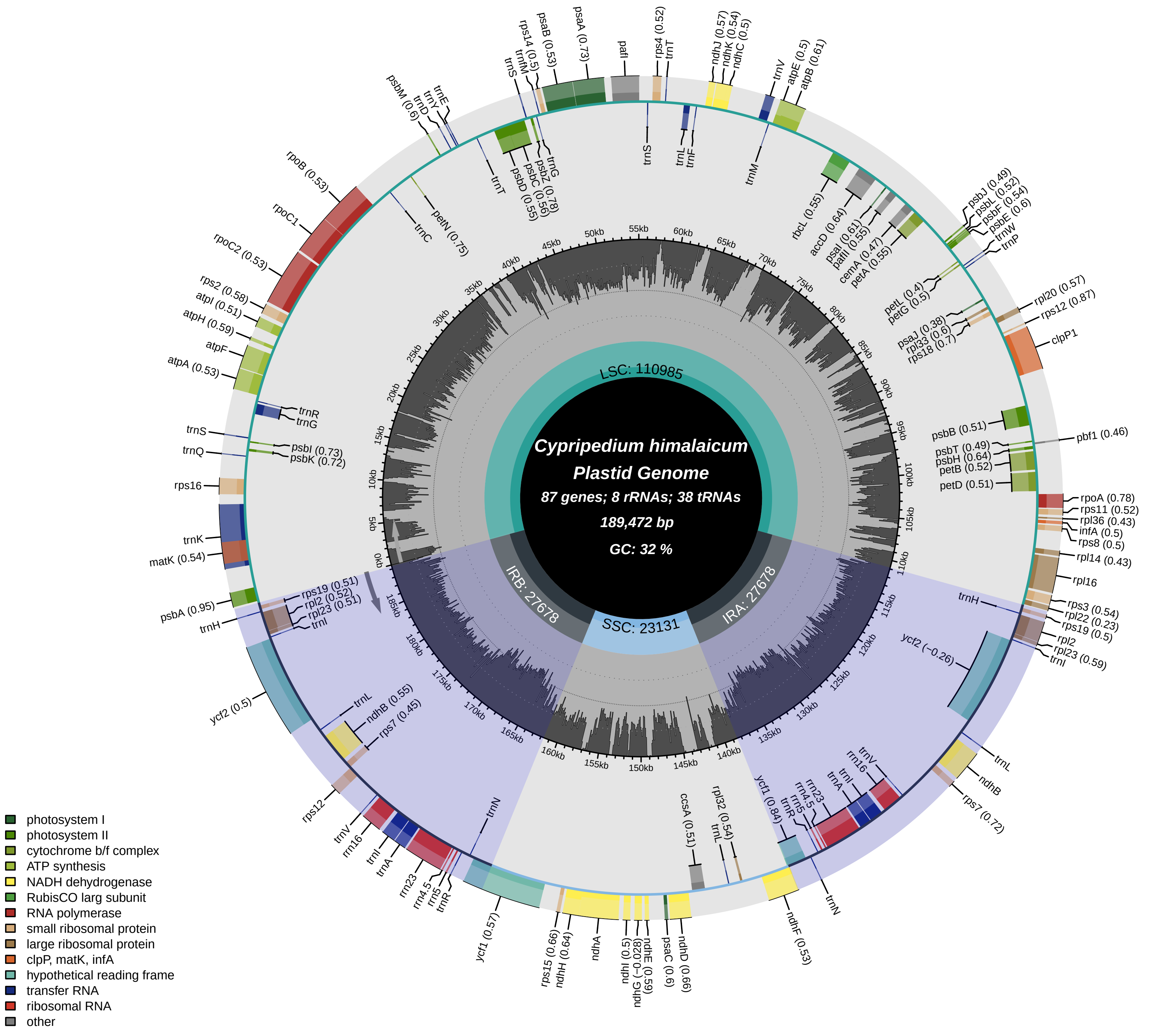
Cypripedium himalaicum
Plastid Genome
87 genes; 8 rRNAs; 38 tRNAs
189,472 bp
GC: 32 %
LSC: 110985
IRA: 27678
SSC: 23131
IRB: 27678
psaB (0.53)
psaA (0.73)
pafI
rps4 (0.52)
trnT
ndhJ (0.57)
ndhK (0.54)
ndhC (0.5)
trnV
atpE (0.5)
atpB (0.61)
rps14 (0.5)
trnfM
trnS
psbM (0.6)
trnD
trnY
trnE
rpoB (0.53)
rpoC1
rpoC2 (0.53)
rps2 (0.58)
atpI (0.51)
atpH (0.59)
atpF
atpA (0.53)
trnR
trnG
trnS
trnQ
psbI (0.73)
psbK (0.72)
rps16
trnK
matK (0.54)
psbA (0.95)
trnH
rps19 (0.51)
rpl2 (0.52)
rpl23 (0.51)
trnI
ycf2 (0.5)
trnL
ndhB (0.55)
rps7 (0.45)
rps12
trnV
rrn16
trnI
trnA
rrn23
rrn4.5
rrn5
trnR
trnN
ycf1 (0.57)
rps15 (0.66)
ndhH (0.64)
ndhA
ndhI (0.5)
ndhG (−0.028)
ndhE (0.59)
psaC (0.6)
ndhD (0.66)
ccsA (0.51)
rpl32 (0.54)
trnL
ycf1 (0.84)
ndhF (0.53)
trnN
rrn23
rrn4.5
rrn5
trnR
trnA
trnI
rrn16
trnV
rps7 (0.72)
ndhB
trnL
ycf2 (−0.26)
trnH
rps3 (0.54)
rpl22 (0.23)
rps19 (0.5)
rpl2
rpl23 (0.59)
trnI
rpl14 (0.43)
rpl16
rpoA (0.78)
rps11 (0.52)
rpl36 (0.43)
infA (0.5)
rps8 (0.5)
psbB (0.51)
pbf1 (0.46)
psbT (0.49)
psbH (0.64)
petB (0.52)
petD (0.51)
rpl20 (0.57)
rps12 (0.87)
clpP1
psaJ (0.38)
rpl33 (0.6)
rps18 (0.7)
petL (0.4)
petG (0.5)
psbJ (0.49)
psbL (0.52)
psbF (0.54)
psbE (0.6)
trnW
trnP
rbcL (0.55)
accD (0.64)
psaI (0.61)
pafII (0.55)
cemA (0.47)
petA (0.55)
trnM
trnL
trnF
trnS
trnG
psbZ (0.78)
psbC (0.56)
psbD (0.55)
trnT
petN (0.75)
trnC
photosystem I
photosystem II
cytochrome b/f complex
ATP synthesis
NADH dehydrogenase
RubisCO larg subunit
RNA polymerase
small ribosomal protein
large ribosomal protein
clpP, matK, infA
hypothetical reading frame
transfer RNA
ribosomal RNA
other
