# Supplementary material for: Comparative Analysis of the Chloroplast Genomes of Cypripedium: Assessing the Roles of SSRs and TRs in the Non-Coding Regions of LSC in Shaping Chloroplast Genome Size
Source: Int J Mol Sci. 2025 Apr 14;26(8):3691. doi: 10.3390/ijms26083691 (PMC12027508; doi:10.3390/ijms26083691)
Supplement: Supplementary file 1 [file C. shanxiense.pdf › C. shanxiense.pdf]

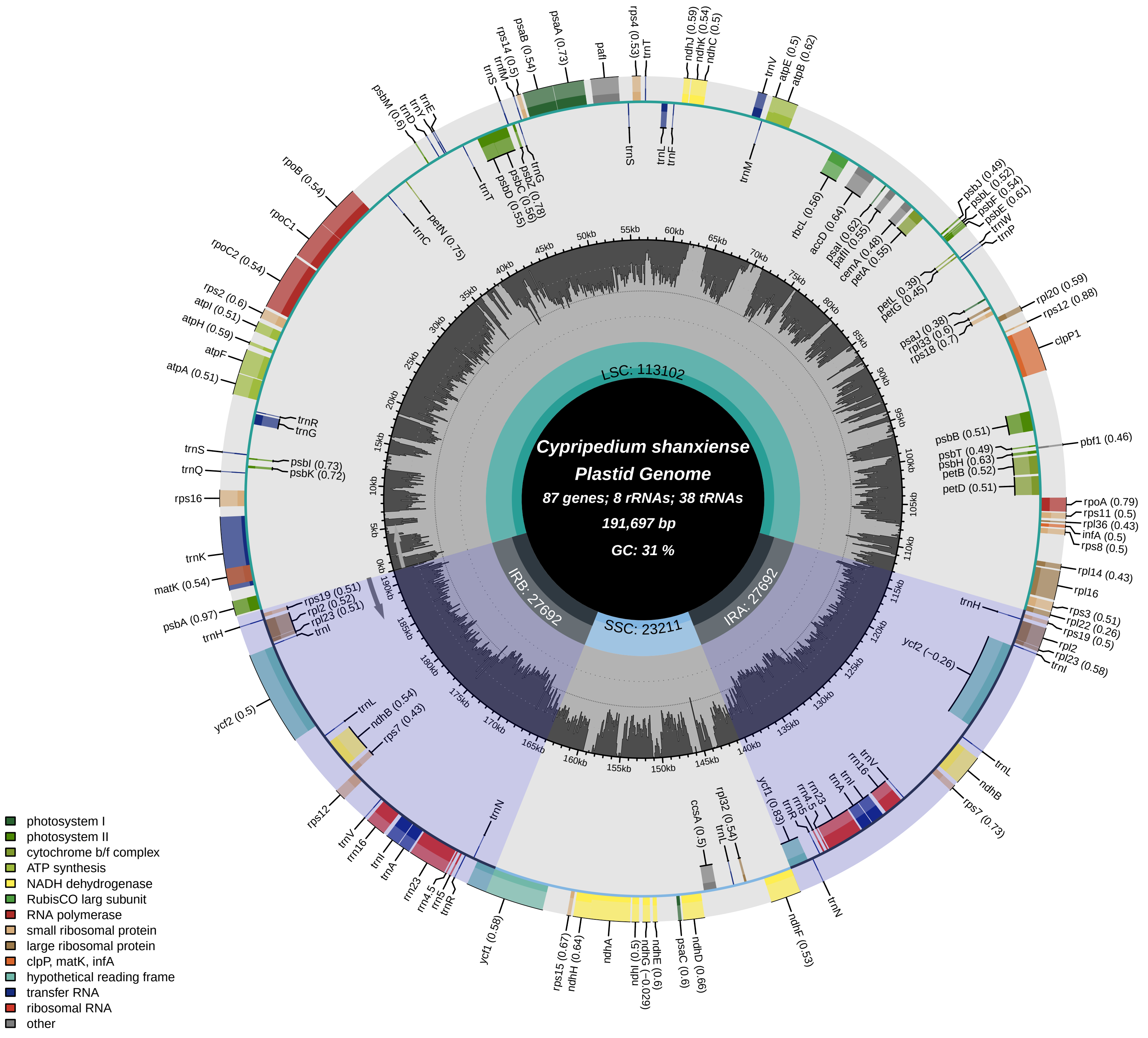
Cypripedium shanxiense
Plastid Genome
87 genes; 8 rRNAs; 38 tRNAs
191,697 bp
GC: 31 %
LSC: 113102
IRB: 27692
SSC: 23211
IRA: 27692
photosystem I
photosystem II
cytochrome b/f complex
ATP synthesis
NADH dehydrogenase
RubisCO larg subunit
RNA polymerase
small ribosomal protein
large ribosomal protein
clpP, matK, infA
hypothetical reading frame
transfer RNA
ribosomal RNA
other
psbA (0.97)
matK (0.54)
trnK
rps16
trnQ
trnS
psbI (0.73)
psbK (0.72)
trnR
trnG
atpA (0.51)
atpF
atpH (0.59)
atpI (0.51)
rps2 (0.6)
rpoC2 (0.54)
rpoC1
rpoB (0.54)
trnC
petN (0.75)
psbM (0.6)
trnD
trnY
trnE
trnT
psbD (0.55)
psbC (0.56)
psbZ (0.78)
trnG
trnS
trnfM
rps14 (0.5)
psaB (0.54)
psaA (0.73)
pafI
rps4 (0.53)
trnT
trnS
trnL
trnF
ndhJ (0.59)
ndhK (0.54)
ndhC (0.5)
trnM
trnV
atpE (0.5)
atpB (0.62)
rbcL (0.56)
accD (0.64)
psaI (0.62)
pafII (0.55)
cemA (0.48)
petA (0.55)
psbJ (0.49)
psbL (0.52)
psbF (0.54)
psbE (0.61)
trnW
trnP
petL (0.39)
petG (0.45)
psaJ (0.38)
rpl33 (0.6)
rps18 (0.7)
rpl20 (0.59)
rps12 (0.88)
clpP1
psbB (0.51)
pbf1 (0.46)
psbT (0.49)
psbH (0.63)
petB (0.52)
petD (0.51)
rpoA (0.79)
rps11 (0.5)
rpl36 (0.43)
infA (0.5)
rps8 (0.5)
rpl14 (0.43)
rpl16
trnH
rps3 (0.51)
rpl22 (0.26)
rps19 (0.5)
rpl2
rpl23 (0.58)
trnI
ycf2 (−0.26)
trnL
ndhB
rps7 (0.73)
trnV
rrn16
trnI
trnA
rrn23
rrn4.5
rrn5
trnR
trnN
ycf1 (0.83)
ndhF (0.53)
rpl32 (0.54)
trnL
ccsA (0.5)
ndhD (0.66)
psaC (0.6)
ndhE (0.6)
ndhG (−0.029)
ndhI (0.5)
ndhA
ndhH (0.64)
rps15 (0.67)
ycf1 (0.58)
trnN
trnR
rrn5
rrn4.5
rrn23
trnA
trnI
rrn16
trnV
rps12
rps7 (0.43)
ndhB (0.54)
trnL
ycf2 (0.5)
trnH
rps19 (0.51)
rpl2 (0.52)
rpl23 (0.51)
trnI
